# Phenolic Compounds and Derivatives in Ruminant Meat and Milk: A Systematic Review

**DOI:** 10.1021/acs.jafc.5c06118

**Published:** 2025-11-14

**Authors:** Muhammad Ahsin, Sulaiman K. Matarneh, Kara J. Thornton, Scott Kronberg, Mamoona Amir, Stephan van Vliet

**Affiliations:** † Department of Nutrition, Dietetics and Food Sciences, College of Agriculture and Applied Sciences, 4606 Utah State University, Logan, Utah 84322, United States; ‡ Department of Animal, Dairy, and Veterinary Sciences, 4606 Utah State University, Logan, Utah 84322, United States; § USDA-Agricultural Research Service, Mandan, North Dakota 58554, United States; ∥ Department of Animal Food Products Technology, Faculty of Food Science and Nutrition, 66927 Bahauddin Zakariya University, Multan, Punjab PK 60800, Pakistan

**Keywords:** metabolomics, phenolics, phytonutrients, meat, milk, grass-fed, pasture-finished

## Abstract

This review synthesizes evidence on phenolic concentrations and diversity in ruminant meat and milk, considering animal species, management, forage, seasonality, and analytical methods. From 39 studies, 356 distinct phenolics were identified in meat and milk, including several from medicinal and non-staple-forage plants. Goat milk showed the highest concentrations as measured by total phenolic content assays (1390 μg GAE/mL) and targeted mass spectrometry (26.79 μg/mL). Beef had the greatest diversity (164 metabolites), followed by sheep milk (110 metabolites); however, beef is also most studied. Organic/agroecological versus conventional systems, fresh versus preserved forages, and younger versus mature pastures were generally associated with a higher phenolic content. Among forages, red clover supported greater diversity than chicory, lucerne, or white clover, while maize silage yielded a higher phenolic content than ryegrass silage. Ruminants can act as biological mediators linking soils, plants, and human diets, often resulting in upcycling of phenolic-derived metabolites from plants not consumed by humans. Future research should integrate soil, plant, animal, and food sciences to fully reveal this role and its potential significance to human health.

## Graphical abstract

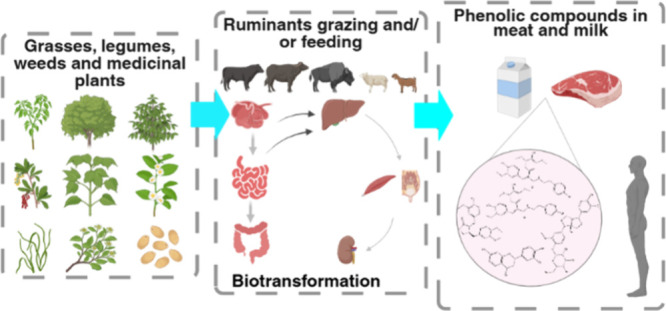

## 1 Introduction

The global market for ruminant-derived foods, including meat and dairy products from cattle, goats, and sheep, has undergone a notable shift, with growing consumer demand for products derived from pasture-raised and grass-fed animals.^1^ This trend is driven by an increased interest in animal welfare, environmental sustainability, and the perceived nutritional benefits of pasture-raised animal products.^2,3^ A growing body of evidence supports these perceptions and indicates that pasture-raised animal products often exhibit a more favorable fatty acid profile, including a lower omega-6 to omega-3 ratio and increased concentrations of long-chain saturated and polyunsaturated fatty acids.^4–7^ Additionally, these products are reported to have higher concentrations of phytochemicals, including terpenoids, carotenoids, phenols, isoflavones, flavonoids, glucosinolates, and their metabolites, which directly reflect in ruminants' diet.^2,5,6^ While the benefits of these phytonutrients for the metabolic health of grazing animals have been systematically reviewed and established,^8–12^ their significance for consumer health remains underexplored.

The understanding of ruminal bioprocessing of phenolics—as they are transferred from plant to animal—has remained an important constraint in advancing research on linking plant-animal-human health.^13,14^ Nonetheless, providing ruminants access to fresh forage and phenolic-rich feed enhances their oxidation status, indicating that these nutrients are transferred into the animal and remain bioactive after digestion.^2,6^ Moreover, these phenolics—and their subsequent mammalian metabolites—have been reported to enrich the animals’ meat and milk.^2,12,15^ For example, goat feed supplemented with *Acacia artesian* pods increased the antioxidant status of the animals and phenolics in their milk,^15^ while incorporation of the phenolic-enriched goat milk in a high-fat diet fed to mice improved their glucose tolerance while preventing adipose tissue hypertrophy and hepatic steatosis compared with mice fed a high-fat diet and control milk not further enriched in phenolics.^16^ In a human trial, consuming pecorino cheese—made from milk of sheep grazing Sardinian mountain pastures—for 10 weeks decreased circulating inflammatory markers.^17^ These findings suggest that phenolics can move up the trophic ladder through animal-based products, while preserving their functional properties.

Analytical sensitivity has struggled to keep pace with these trace-level compounds, creating a persistent technological bottleneck;^18^ however, modern mass spectrometry (MS) platforms have begun to overcome limitations in analytical sensitivity. Orbitrap high-resolution accurate mass (HRAM) analyzers can capture thousands of ions per scan with exceptional signal-to-noise ratios, allowing the annotation of trace compounds such as phenolics in complex matrices such as meat and milk.^19,20^ Modern triple quadrupole (QQQ) instruments provide up to six orders of linearity and achieve low picogram/mL detection limits, with dwell times as short as 5 ms or less, thereby enabling the monitoring of hundreds of transitions within a single run of a few minutes.^21,22^ Thus, when used together—Orbitrap HRAM for discovery and QQQ for quantitation—these tools provide the sensitivity and dynamic range to better understand the flow of phenolics from forage to ruminant food products.

Beyond advances in the understanding of ruminal bioprocessing and analytical sensitivity, a critical challenge is reframing phenolics not as “phytotoxins” but as context-dependent nutrients. For example, isoflavonoids—a class of phenolics known as phytoestrogens—have long been in the spotlight because of their association with impaired reproductive performance.^23^ However, recent studies suggest that isoflavonoids can both impair and enhance reproductive function, depending on factors such as dosage, timing, and individual physiology.^24^ Similarly, condensed tannins (CTs) have been studied for their effects on protein digestibility.^25^ Although high levels of CTs can reduce overall protein availability and negatively affect animal performance, moderate levels have been shown to reduce protein degradation in the rumen, thereby increasing the flow of undegraded protein to the intestine and improving amino acid absorption.^25,26^ Beyond these specific compounds, phenolic research has traditionally focused on plants with medicinal value^27^ and forages capable of promoting animal health.^3,28^ Nonetheless, the potential of phenolic-rich animal-derived foods to serve as secondary dietary sources of phenolics for humans has received comparatively less attention.^2,29^

Ruminant grazing behavior is guided by their nutritional demands and the health benefits associated with phenolics in various plants.^30,31^ Typically, animals rely on two to three plants to support their growth, development, and metabolic needs, while supplementing their diet with smaller portions of minor plants, which are often selected prophylactically and medicinally if animals are “locally adapted” and familiar with these plants.^32^ Research has found that animals forage differently depending on their physiological and health conditions, relying on orosensory feedback, such as satiety or malaise.^3^ This suggests that animals utilize the plethora of phenolics present in different plants to improve their health and resilience. Additionally, because many of the plants consumed by ruminants are indigestible to humans, ruminants play a unique ecological role in introducing both additional and unique forage-derived phenolics into the human diet.

This is exemplified by Reynaud et al.,^33^ who studied 24 permanent pastures and identified 31 different phenolics across 90 plant species, the majority of which are not consumed by humans. Additionally, a recent systematic review further highlights the extensive phenolic diversity in forage plants.^34^ The authors studied 27 different plant species, including grasses, forbs, legumes, and brassicas, which collectively contained 488 different phytochemicals. Phytochemical diversity also varied among plants, ranging from phytochemically diverse species such as *Cichorium intybus* (chicory) with 92 compounds and *Trifolium repens* (white clover) with 125 compounds, to more modest profiles with only 4 compounds annotated in *Lotus pedunculatus* (lotus major) and*Phacelia tanacetifolia* (Phacelia), reflecting the unique phytochemical profiles of different forage species. It must be noted that the latter plants are likely to contain many more phytochemicals; however, their phenolic makeup may not yet be cataloged properly.

Studies have reported that diverse pasture grazing increase plasma antioxidant capacity, decreases lipid peroxidation, and improves immune function in cattle, sheep, and bison compared with monotonous pastures,^35,36^ explained by the diverse pool of phenolics.^12^ Additionally, skeletal muscle of animals grazing on fresh forages tends to have a greater reliance on oxidative metabolism, indicating improved mitochondrial function,^4^ which arguably further reflects enhanced metabolic health compared with feedlot-fed animals. This observation may be the result of greater exposure to long-chain polyunsaturated fatty acids and/or greater physical activity.^12^ Certain fresh forages have also been associated with improved biomarkers of behavioral well-being,^3,35^ increased growth hormone levels,^37^ and reduced methane emissions in the case of tannins.^38^ Moreover, they appear to potentiate anti-inflammatory,^39^ wound healing,^40^ antiparasitic,^40^ immune,^40^ and antimicrobial responses in livestock.^39^ Collectively, this evidence suggests that animals grazing on diverse and fresh forages harvest a wider pool of phenolics, contributing to improvements in their health and welfare. Additionally, their products (e.g., meat and milk) are also enriched in these bioactives and may serve as secondary sources of phenolic-derived metabolites for humans.^41,42^ For instance, goat milk has been reported to provide up to 334 mg GAE phenolics per serving,^43^ which is comparable to 320 mg GAE per serving of some green teas, although the individual compounds the provide would be very different.^44–46^ However, it should be noted that the direct consumption of plant foods (e.g., fruits, vegetables, whole grains, and legumes) remains the predominant source of phenolics in the human diet. The phenolic metabolites from animal-sourced foods, often bioprocessed into mammalian metabolites, should therefore be regarded as complementary rather than substitutive to plant food consumption, given their distinct metabolome profiles.^47^

Significant progress has been made at the landscape and societal levels^8,48^ and from chemoscape perspectives,^9^ including phenolic-rich pasture designs;^10^ however, phenolic profiling of major forage plants consumed by rumants^34^ and mapping health information regarding phenolic profiles of ruminant-sourced foods and potential human health benefits remains limited.^2^ Furthermore, compiling and harmonizing the analytical methods used to profile these compounds is essential for advancing standardized protocols in future research.

Accordingly, this systematic review aimed to compile the existing literature on phenolics in meat and milk from cattle, buffalo, bison, sheep, and goats and generate a concise inventory of phenolics identified in these animal-sourced foods, along with their links to feed composition, seasonality, and management practices. Upon beginning, a preliminary search revealed that only a fraction of the available information about feed composition had been systematically examined. Moreover, no standardized protocols have been established for the extraction and analysis of these compounds. Interestingly, aside from one study that screened 203 phenolic metabolites and successfully quantified 25 compounds,^49^ almost none of the existing studies were designed with phenolic profiling of animal-derived products as the primary objective. In addition, the limited availability of quantitative data—further complicated by heterogeneous sample processing methods—hampers comparisons. Large variations between animal breeds, limited reporting on animal feed, and diverse analytical methodologies make cross-study comparisons of quantitative data particularly challenging. Therefore, the objective of this work was: (i) to systematically review the literature on phenolics reported in ruminant food products, relate those phenolics back to plant sources where possible, and assess their potential benefits for animals and humans; and (ii) to provide methodological guidance for future studies, including a list of compounds that could be prioritized in targeted metabolomics assays.

## 2 Materials and Methods

A comprehensive literature search was conducted following the Preferred Reporting Items for Systematic Reviews and Meta-Analyses (PRISMA) guidelines.^50^ Separate searches were performed in four major databases: PubMed, Scopus, the Directory of Open Access Journals (DOAJ), and Google Scholar. To ensure the inclusion of maximum data, forward and backward citation searches were conducted on Google Scholar and in seven key journals that appeared in the primary database searches, *Meat Scienc*e, *International Dairy Journal*, *Metabolites*, *Foods*, *Food Chemistry*, *Animals*, and *Journal of Dairy Science*, as suggested best practice.^51^ All data were sourced from peer-reviewed journal articles. The following search terms were used: (“milk” OR” meat”) AND (“phytoch” OR “secondary metabol” OR “secondary com” OR “health” OR “alkal” OR “coum” OR “pheno” OR “flav” OR “lign” OR “terpen” OR “sapon” OR “stilbe” OR “tann” OR “quin”) AND (“chromatography” OR “mass spec” OR “MS” OR “metabolom” OR “HPLC”). Eligible studies were published after January 2000 and were in English. Titles and abstracts of unique studies were screened for reports on phenolics (either qualitative or quantitative data) in the meat and milk of cattle, buffalo, sheep, goats, and bison, including the presence or concentration of phenolics in meat and/or milk. Studies using in vitro models, rumen-protected phenolic supplements, and intraintestinal infusion models were excluded. Data extraction was performed by the leading author (MA) and independently reviewed by all coauthors to ensure accuracy and consistency. All information extracted from each study is summarized in Table S2. The quality of data reporting was assessed using a tailored protocol developed from the Systematic Review Center for Laboratory Animal Experimentation (SYRCLE) risk of bias tool.^52,53^ Comprehensive details of the literature search and selection process followed for this systematic review are provided in Supporting Information Figure S1.

## 3 Results and Discussion

### 3.1 Study Inclusion

The relevant data extracted from the selected studies are presented in Table S2. The investigations primarily focused on how different diets (e.g., fresh forages vs total mixed rations), farming practices (e.g., organic vs conventional pastures), seasons (e.g., dry vs rainy seasons), and breed/species influence specific phenolic classes in meat and milk. The study designs included 19 observational studies and 20 controlled feeding trials, reflecting a diverse range of research methodologies. Geographical region analysis showed the highest contributions of literature from Europe, including 12 from Italy, two each from the UK, Finland, Norway, and Denmark, and one each from France, the Czech Republic, Sweden, Switzerland, Greece, and Hungary. Contributions from North America included five from the USA and one each from Canada and Mexico, while Asia included four from China and one from Israel, and Africa included one study from Kenya. Studies from Europe and Asia predominantly examined milk, whereas those from North America focused on meat.

### 3.2 Risk of Bias and Quality of Reporting Assessment

Among the selected studies, 59% demonstrated low selection bias, while 24% exhibited high selection bias, as evaluated using the SYRCLE risk of bias tool (Figure S2a,b).^52,53^ Measurement bias was low in 77% of the studies, whereas confounding bias (e.g., due to uncontrolled variables such as animal sex, age, feed intake, and pasture quality) was high in 22% of the studies. For instance, Bennato et al.^54^ reported that the quantification of phenolics in sheep milk is potentially at high risk of measurement bias due to incomplete reporting on internal standards, extraction recovery/efficiency, and calibration/linearity (e.g., *R* ^2^). The risk of selective reporting bias was high in only 18% of the studies included. These findings underscore the importance of addressing these biases to enhance the reliability and validity of the research outcomes. Most of the selected articles clearly stated their research objectives and hypotheses (91%), employed appropriate study designs (88%), applied suitable statistical methods (82%), and provided data on the variability (97%). However, reporting on conflicts of interest, ethical approval, and method validation was notably low, at 38, 44, and 41%, respectively. These gaps highlight the need for improved transparency and methodological rigor to strengthen the quality and credibility of future studies.

### 3.3 Extraction Solvents for Meat and Milk Phenolics

Various solvents have been employed in different studies for the extraction of metabolites, including phenolics, with each solvent influencing the migration and recovery of compounds based on their affinities.^55^ Commonly used solvents include acetonitrile, methanol, ethanol, water, and mixtures thereof. For example, Rocchetti et al.^56^ utilized 0.1% formic acid in acetonitrile for sheep milk, whereas Bennato et al.^54^ employed a 70:30 (v/v) ethanol: water solution. In contrast, Hernandez et al.^57^ extracted meat using a 50:50 (v/v) methanol: water solution, and lipids were removed using chloroform. In contrast, Evans et al.^4^ and Ahsin et al.^12^ extracted meat samples in methanol without removing lipids from the samples. The choice of extraction solvent is also likely to impact the results of more crude assays, such as the total phenolic content (TPC) assay. Mokrani and Madani^58^ reported a higher phenolic extraction efficiency of acetone compared with methanol; however, the methanol extract showed significantly higher flavonoids (a class of phenolics) than acetone. Of note, Kasapidou et al.^43^ reported the highest TPC content (1390 GAE/mL milk) among all meat and milk samples and used an acetonitrile solution with 4% acetic acid for extraction, while Di-Grigoli et al.^59^ extracted sheep meat total phenolics in distilled water and reported up to 720 μg GAE/mL milk. Nonetheless, these studies underscore the importance of standardizing the extraction solvent to eliminate bias, thereby enabling more accurate comparisons across variables such as animal species, farming practices, and feed types.

### 3.4 Phenolics Quantitation Method for the Ruminant Meat and Milk

The TPC assay is a widely used method for quantifying phenolics in biological samples, including meat and milk.^43,59–66^ The assay is based on the reaction of phosphomolybdic and phosphotungstic acids with phenolic hydroxyl groups under alkaline conditions, resulting in the formation of blue molybdenum–tungsten oxides.^67^ The intensity of the color formed is directly proportional to the concentration of phenolic groups and measured spectrophotometrically with the results expressed as gallic acid equivalents (GAEs). Among the 39 articles reviewed, nine reported TPC in meat and/or milk (Figure 2); however, the interest in using TPC assays has declined in recent years, partly due to methodological concerns^68^ and because of the growing emphasis on annotating and quantifying individual phenolics in food. The latter shift is driven by the recognition of health benefits associated with different phenolics, and establishing structure–activity and quantitation requires advanced profiling techniques.^68–70^

The advent of liquid chromatography (LC) has enabled the individual profiling of phenolics in various foods, particularly when coupled with UV–Vis spectrophotometry or MS. LC separates compounds based on their interactions with the mobile and stationary phases, thereby allowing individual nutrients to be more accurately profiled and quantified by UV–Vis absorbance.^71^ However, due to the coelution of structurally similar phenolics and their ability to absorb similar wavelengths, typically around 270 nm,^72^ this method is still prone to analytical artifacts (quantitative overestimation and qualitative misidentification). Therefore, only four studies included in the present systematic review employed this technique, with the latest study published in 2009,^73–76^ potentially indicating a lower interest from the scientific community in employing this technique. In contrast, LC coupled with tandem mass spectrometry (MS/MS) can differentiate structurally similar compounds based on their ion mass-to-charge (*m*/*z*) ratios and fragmentation patterns.^77^ This multilayered filtering greatly improves specificity and confidence in the identification of structurally similar compounds like phenolics.^78^ Twenty-five studies in this review utilized LC-MS/MS, with the latest study being from 2025, highlighting its widespread use for phenolic profiling in recent times.

MS/MS assays are divided into qualitative (untargeted metabolomics) and quantitative (targeted metabolomics) approaches. Untargeted metabolomics can detect thousands of metabolites, but its performance is limited by its lower sensitivity (compared with the targeted approach) and the coverage of spectral libraries.^49,79^ For example, Wang et al.^80^ detected 2561 unique metabolites in goat meat and accurately annotated 529 of them, of which 44 were phenolics. Hernandez et al.^57^ identified 119,957 unique spectral features in cattle meat, but were only able to annotate 377, among which 117 were phenolics. These findings highlight that spectral library coverage is a major bottleneck in annotation. Forage phytochemical profiling faces the same challenge; for example, Reynaud et al.^33^ sampled pasture with 90 plant species, detected 92 distinct peaks, and were able to annotate only 31 phenolics.

On the other hand, targeted metabolomics offers more sensitivity and accuracy (compared with the untargeted approach), and when mixtures of purified standards are used, this approach enables the quantification of metabolites; however, the number of metabolites that can be quantified is limited by the availability of purified standards. To overcome the constraint of the availability of authentic standards, some studies employ semi-targeted approaches that often use a parent phenolic compound as a calibration standard to estimate the concentration of all phenolics. However, results show that quantifying phenolic metabolites by referencing their unmetabolized parent compounds can result in both underestimations (up to 94%) and overestimations (up to 113%) of individual metabolite concentrations, due to differences in ionization efficiency and detector responses between parent and metabolite forms.^81^ For example, Agulló et al.^49^ scanned for 203 phenolic compounds in cow and goat milk—the highest number among all studies in this review—but were only able to quantify 26. Although untargeted assays are less sensitive, this approach was able to annotate up to 117 phenolics in meat and milk, whereas targeted assays currently published, identified a maximum of 26. This discrepancy may reflect biological variation, limitations in compound selection, and potential misclassification in untargeted approaches. Additionally, untargeted approaches do not allow for quantitation and are limited to determining the relative differences. Therefore, accurate quantitation is also important to establish thresholds of intakes with a potential biological significance to consumers. Nonetheless, combining both targeted and untargeted methods is essential for a comprehensive understanding of the metabolic landscape, balancing precise quantification with broad-spectrum discovery.

Gas chromatography (GC) is commonly used to separate volatile compounds and can also be coupled with MS/MS.^82^ However, because phenolics are largely nonvolatile,^83^ only one study employed GC-MS/MS using an untargeted metabolomics approach. The study reported a single phenolic-related compound, hippuric acid,^84^ indicating that GC-based approaches have limited application.

Fourier-transform infrared spectroscopy (FTIR) is a nondestructive technique used to identify and quantify food components based on their infrared absorption spectra.^85^ However, among the selected articles, only one study employed FTIR for untargeted metabolomics, identifying a single phenolic compound—hippuric acid—in cow’s milk.^86^ The limited application of FTIR in the phenolic analysis of animal-based foods may be attributed to the complex nature of the tissue matrix and low concentrations of phenolics, which make detection and quantification challenging.^87^ In summary, the choice of analytical method for phenolic determination depends on the specific research objectives, with each technique offering distinct advantages and limitations. However, the integration of untargeted and targeted LC-MS/MS assays currently offers the most robust and detailed phenolic profiling capabilities. Meanwhile, traditional methods, such as TPC assays and FTIR, may remain valuable for specific applications, particularly in rapid screening and low-cost analysis; however, their inaccuracies should be noted.

### 3.5 Metabolic Fate of Plant Phenolics in Ruminants

The absorption of phenolics and other phytonutrients in ruminants is influenced by molecular weight, biochemical state (such as glycosylation, esterification, and methylation), and concentrations in consumed plant (Figure 1).^88^ Rumen anaerobic microbes transform dietary phenolics via three biochemical pathways: (i) side-chain hydrogenation to saturated 3-phenylpropionate derivatives,^89^ (ii) O-demethylation of methoxy groups into free phenolic hydroxyls;^90^ and (iii) reductive dihydroxylation of aromatic rings to simpler aromatic molecules.^91^ In vivo models show that cinnamic acids (a major class of simple phenols) undergo rumen microbial biohydrogenation of the side chain to 3-phenylpropionic acid, followed by dehydroxylation to 2- and 3-hydroxy-3-phenylpropionic acids.^92^ Rumen O-demethylation of isoflavonoids formononetin and biochanin A to daidzein and genistein, respectively, is well documented in vivo and in vitro*.* ^93,94^ Flavonoids are also metabolized in the rumen; for example, quercetin is transformed to 3,4-dihydroxyphenylacetic acid and 4-methylcatechol via ring cleavage and dihydroxylation. Thus, the rumen microbiota metabolizes dietary phenolics via hydrogenation, O-demethylation, and reductive dehydroxylation, yielding lower-molecular-weight derivatives (e.g., phenylpropionic-, phenylacetic-, and hydroxybenzoic acids) with greater hydrophilicity and absorption potential.^92^

**Figure 1:**
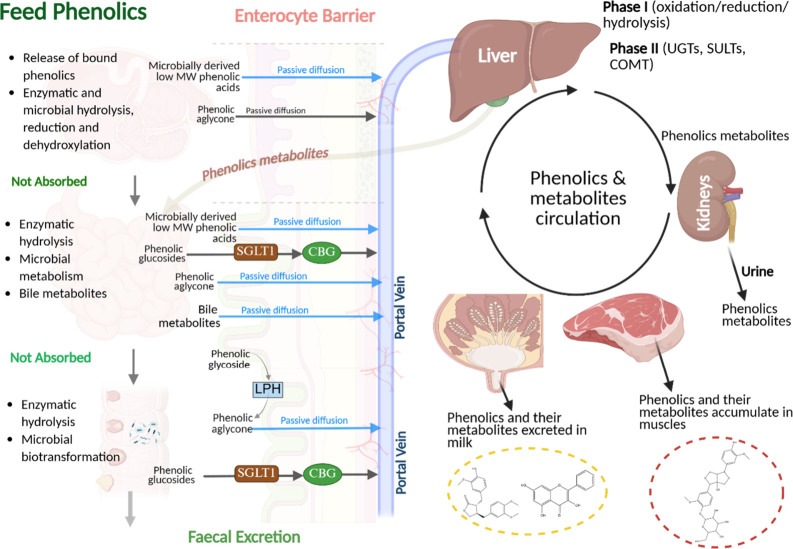
Model of in-depth pathway analysis of phenolic compounds as they flow from plants to ruminant digestion to tissue (meat) and milk. The biochemical processes these compounds undergo in the rumen and small intestine, routes of absorption through enterocyte barriers, metabolism in the liver and kidney, and transfer pathways into muscle and milk. SGLT1 = sodium-glucose transport, CBG = corticosteroid-binding globulin, and LPH = lactase-phlorizin hydrolase, UGTs = UDP-glucuronosyltransferases, SULTs = sulfotransferases, and COMT = catechol O-methyltransferase. The figure was created with biorender.com.

In sheep, ruminal cinnamic acid infusion increased 3-phenylpropionic acid concentrations dose-dependently, with 70–105% of the dose recovered in urine as benzoic/hippuric acid, reflecting microbial conversion and enhanced systemic availability.^92^ In dairy cows, intraruminal quercetin rapidly degraded to 3,4-dihydroxyphenylacetic acid and 4-methylcatechol, which were subsequently detectable in plasma and urine.^95,96^ Unlike monogastric animals, ruminants hydrolyze proanthocyanidin polymers to bioactive monomers (e.g., catechin/epicatechin) via rumen microbes, improving bioavailability of otherwise poorly absorbed polymers.^97^ Collectively, these findings indicate that the rumen substantially metabolizes phenolics and that downstream metabolites, as opposed to the parent compounds found in plants, are generally the predominant forms entering circulation. Rumen enzymes exhibit higher activity than in monogastric intestines, suggesting greater ruminal capacity for plant phenolic extraction and utilization.^98,99^ Additionally, luminal hydrolysis by lactase-phlorizin hydrolase, located on the brush border of the small intestine, can cleave various flavonoid glycosides, enabling their subsequent absorption by passive diffusion.^100^ Since plant phenolics mainly exist as poorly absorbed esters and glycosides, the predominance of ruminal metabolites in circulation emphasizes the vital role of ruminal microbial esterases and β-glucosidases in their biotransformation, thereby enhancing bioavailability in ruminants

After absorption, phenolics undergo phase I metabolism (oxidation, reduction, hydrolysis) and phase II metabolism (conjugation via glucuronidation, sulfonation, methylation), catalyzed by UDP-glucuronosyltransferases (UGTs), sulfotransferases (SULTs), and catechol-O-methyltransferases (COMT).^101,102^ These processes enhance hydrophilicity, aiding systemic transport and renal excretion—e.g., converting 3-PPA to hippuric acid. Phenolic metabolites circulate in the body and tissues for hours to days postingestion, varying by chemical form and dietary source, before removal via urine or bile.^103,104^ Bile-excreted conjugates may undergo gut deconjugation and reabsorption (enterohepatic recirculation), extending their apparent half-life compared with renal-excreted forms.^105^ Importantly, phenolic hydroxyl groups often remain intact, enabling metabolites to retain or enhance health benefits over parent compounds.^106^ Since phenolic metabolites exhibit diverse physicochemical and biological properties, transformations in the rumen, colon, intestinal, and hepatic enterocytes determine their solubility, bioavailability, and activity.^107^ Thus, research must prioritize analyzing actual phenolic metabolite forms and conjugation patterns in animals, rather than feed-intake estimates or parent plant compounds alone, to elucidate mechanisms, quantify biological value, and predict impacts on animal health and phenolic content in meat and milk.

### 3.6 Phenolic Concentrations in Ruminant Milk

Of the 39 articles in this systematic review, 31 provided quantitative data on milk phenolics (Table S2). Eight reported TPC, while 16 quantified 1–25 individual phenolics. Cow’s milk was most studied (20 publications), followed by goat and sheep (9 each), and buffalo (1). Since TPC and targeted-sum approaches are incomparable and targeted totals vary by compounds included, we avoided pooling totals across studies. For example, in goat milk, the lowest targeted total (0.01 μg/mL) came from UK commercial samples analyzed for five compounds (daidzein, genistein, enterolactone, matairesinol, glycitein),^108^ whereas the highest (26.79 μg/mL) was from Italian pasteurized whole milk using a 25-compound panel, with hippuric acid comprising 88%.^49^ The UK study did not measure hippuric acid, making direct total comparisons invalid; however, both measured enterolactone, with the UK value being 49% higher, highlighting how panel composition—not inherent matrix concentration—may drive apparent differences. Targeted MS assays for total phenolic metabolites in meat and milk are limited by fewer compounds in the panel, contributing to lower values than TPC assays (though they are also not directly comparable).^68^ As targeted MS methodologies improve, more compounds will arguably be quantified, yielding higher total estimates and likely reducing the reliance on a few major metabolites in targeted panels (e.g., hippuric acid). Thus, we focus on within-study comparisons and summarize findings by the assay type.

#### 3.6.1 Cow Milk

TPC in cow’s milk ranged from 25.0 to 58.3 μg GAE/mL and was reported only in two studies (Figure 2).^65,66^ Leparmarai et al.^65^ investigated the effects of urea supplementation, seasonal variations, and cattle breed differences—specifically indigenous Pokot cows and crossbred cows (a mix of East African Boran *Bos indicus* and Guernsey *Bos taurus* )—in semiarid rangeland settings, while the other study measured TPC in commercial milk samples from Holstein and Simmental breeds.^65^ In the first study, urea supplementation during the transition period—between the rainy season and the main dry season—resulted in a 10% decrease in TPC across both breeds; however, during the rainy season, TPC increased by approximately 5% in the crossbred cows but decreased by about 26% in Pokot cows with urea supplementation. The authors attributed the decrease in TPC with urea supplementation to reduced forage intake in the Pokot breed.^65^ On average, milk produced during the rainy season had ∼81% higher TPC than milk from the transition period, and Pokot milk exhibited ∼7% higher TPC than crossbred milk. It was hypothesized that the higher TPC in Pokot milk during the rainy season potentially reflects the breed’s greater efficiency in utilizing local forages. In the second study, Holstein milk had 14% higher TPC than Simmental milk (42 μg GAE/mL), with concentrations comparable to those in the first study during the transition period;^66^ however, as this study analyzed commercial samples, any differences between breeds could also be related to difference in feed.

**Figure 2:**
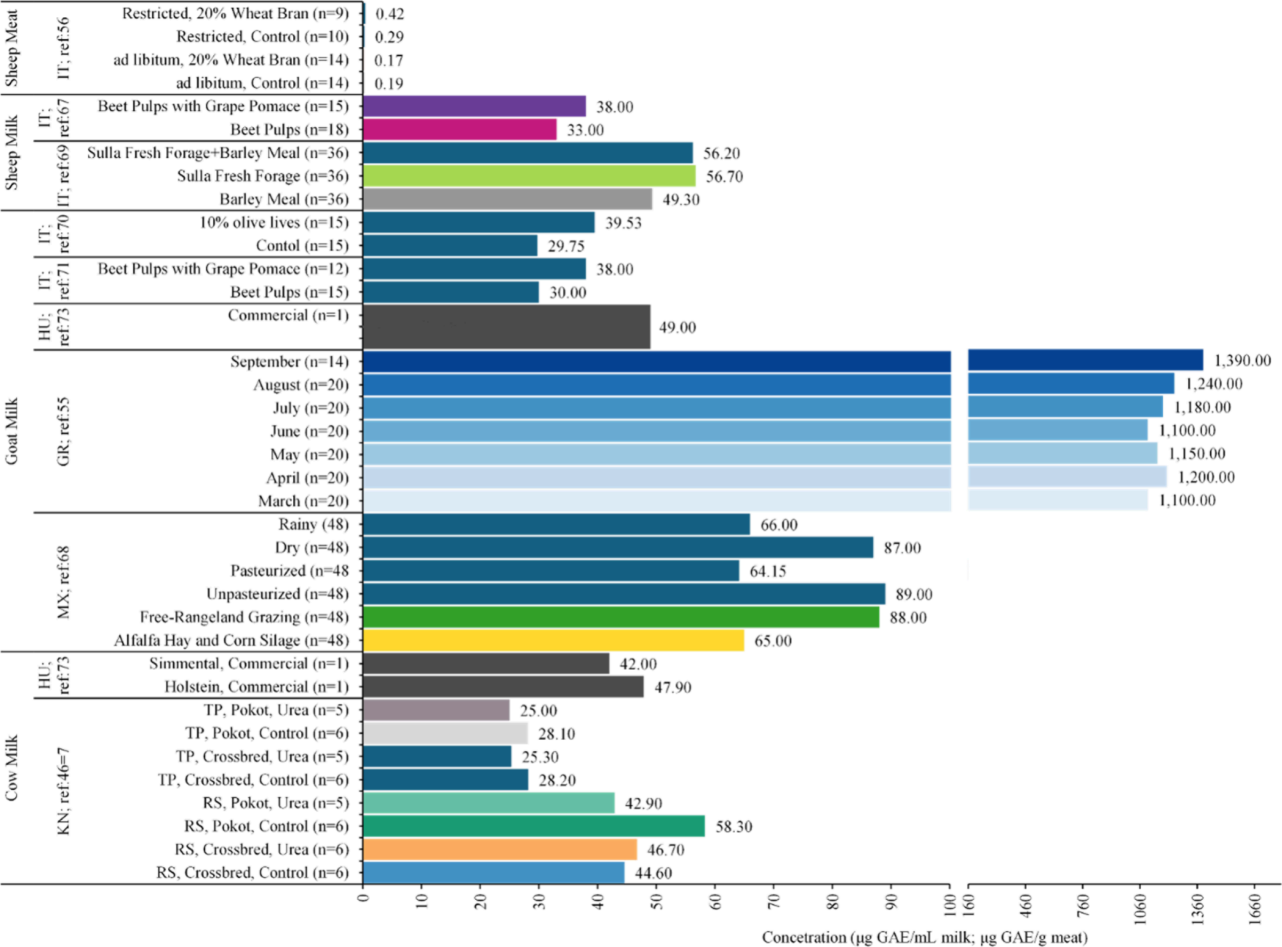
Total phenolic concentration (TPC) in milk and/or meat from the included studies, organized by experimental groups as reported in the original studies. Values are group means; *n* denotes the number of biological replicates per group. ‘ref:’ shows the reference of the study, and country names are given in International Organization for Standardization (ISO) 3166-1 alpha-2 codes.

Studies utilizing targeted phenolic assays in cow milk revealed concentrations ranging from ∼50 to 16.33 μg/mL, with variation driven by diet (e.g., oilseed supplements, silage, hay, concentrate-rich rations, and pastures), season, management, processing (pasteurized vs raw, skim vs whole, etc.), and—perhaps most critically—the size and scope of the analytical panel (Figure 3). In a nine-compound panel, milk from animals grazing red clover (0.33 μg/mL) pasture was ∼400% higher than white clover pasture (0.07 μg/mL), ∼317% higher than chicory pasture (0.08 μg/mL), and ∼257% than lucerne pasture (0.09 μg/mL).^73^ Another study, utilizing a panel of 10 compounds, also found higher levels of phenolics from grazing red clover pasture compared with grazing white clover pasture (0.10 vs 0.08 μg/mL).^109^ A separate study with a panel of 11 compounds reported ∼283% higher phenolics in milk from a second cut birdsfoot trefoil pasture than a third cut clover pasture.^110^ Collectively, these results indicate that forage type leads to differences in milk phenolics, with the highest levels found in animal grazing birdsfoot trefoil, followed by red clover, lucerne, chicory, and white clover. Interestingly, these trends align with TPC values in these plants.^111,112^ However, it is important to highlight that results are limited to a panel of compounds measured, and trends may change with different panels. Additionally, grazing more biodiverse pastures—consisting of mixtures of plant species, may provide a wider variety of phenolics—and, therefore, higher levels in milk compared with grazing more monoculture pastures.^113^

**Figure 3:**
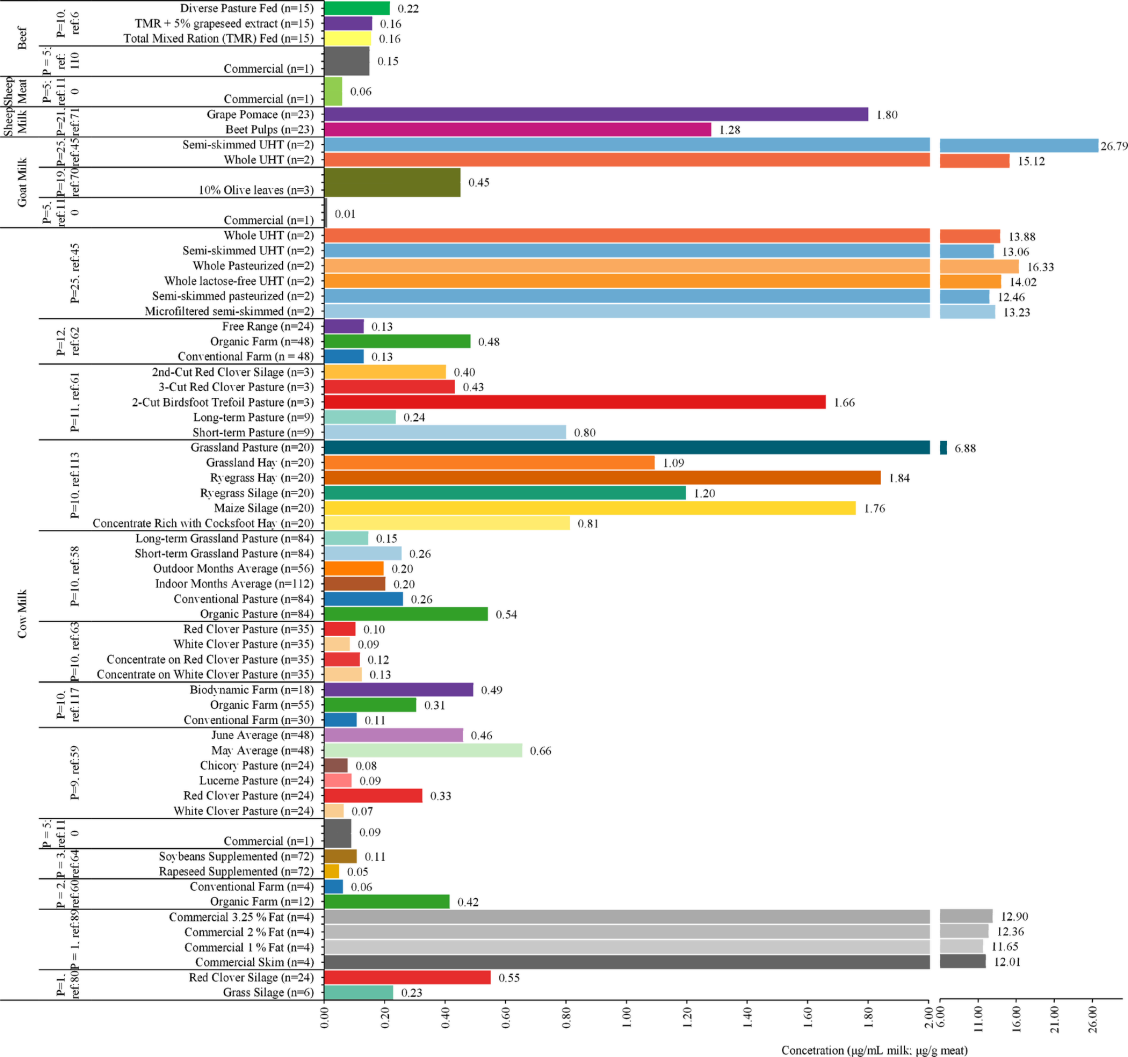
Concentrations of measured phenolics in milk and/or meat from the included studies, organized by experimental groups as reported in the original studies. Values are group means; ‘*P*’ indicates the number of phenolics quantified, ‘ref:’ shows the reference of the study; ‘*n’* indicates the number of biological replicates per group. The study country is denoted by International Organization for Standardization (ISO) 3166–1 alpha-2 codes.

The growth stage of the pasture is another factor that impacts phenolic levels in milk. A panel of 10 compounds found ∼75% higher phenolic levels in milk from young (short-term) grassland pastures (0.26 μg/mL) compared with more mature (long-term) grassland pastures (0.15 μg/mL).^114^ Similarly, a panel of 11 compounds showed ∼238% higher levels in short-term pastures (0.80 μg/mL) compared with long-term pastures (0.24 μg/mL).^110^ Short-term pastures likely provided more phenolic-rich forbs and legumes as well as younger, rapidly regrowing herbage with lower nitrogen conditions. These factors are known to upregulate plant phenylpropanoid pathways,^115^ thereby increasing the transfer of phenolic metabolites into milk—consistent with the ∼75 to 238% higher levels observed. However, these findings do not necessarily imply that long-term pastures are inherently unable to support high phenolic levels; rather, they may reflect site-specific conditions, such as possible overgrazing in the long-term pastures, as examined in the referenced studies.^110,114^

A study with a panel of nine compounds revealed that milk from May (0.65 μg/mL) had 42% higher isoflavonoids compared with June (0.46 μg/mL).^73^ The results suggest that younger pasture plants in May potentially contain higher levels of isoflavonoids, considering the fact that animals were on the same pasture and the same compounds were measured. Another study with a panel of ten compounds reported only 3% higher milk isoflavonoids in indoor months compared with outdoor months.^114^ The results suggest that isoflavonoids are either well preserved or it is more likely that the difference is related to botanical differences in the feed vs forage. Overall, plant phenology and pasture botany appear to dominate over the housing of animals in terms of isoflavonoids in milk. Besides, breed and feed/forage composition processing can also impact phenolic content. Hippuric acid, a major phenolic metabolite in ruminants, measured in commercial 3.25% fat milk was ∼4% higher than in 2% fat milk (12.36 μg/mL), ∼7% higher than in skim milk (12.01 μg/mL), and ∼11% higher than in 1% fat milk (11.65 μg/mL).^86^ In another study analyzing a panel of 25 compounds—the highest number among all products—whole pasteurized milk contained ∼16% more phenolics than whole lactose-free UHT milk (14.02 μg/mL), ∼18% more than whole UHT milk (13.88 μg/mL), ∼25% more than microfiltered semiskimmed milk (13.23 μg/mL) and semiskimmed UHT milk (13.06 μg/mL), and ∼31% more than semiskimmed pasteurized milk (12.46 μg/mL).^49^ Collectively, both studies suggest that phenolic levels vary by up to 31% across commercially available milk products, which may be attributable to differences in milk processing methods.

Similar findings to those described above in dairy cows grazing fresh forages have also been reported in cows fed conserved forages, although seasonality is less likely to play a role. Equol, a metabolite derived from isoflavones, was found at ∼141% higher concentrations in milk from cows fed red clover silage compared with those fed grass silage.^75^ Another study utilizing a 10-compound panel found that ryegrass hay transfers ∼5% (1.84 μg/mL) more phenolics into milk compared with maize silage (1.76 μg/mL), ∼54% more than ryegrass silage (1.12 μg/mL), and ∼68% more than grassland hay (1.09 μg/mL).^113^ Another study using an 11-compound panel found red clover silage (0.40 μg/mL) yielded ∼7% lower phenolics than red clover pasture milk (0.43 μg/mL).^110^ These results indicate that forage hay tends to supply more phenolics than silage but at levels lower than those in fresh forages. This is consistent with previous reports finding that phenolics degrade during the forage conservation process, with the effects being more pronounced in silage production than in hay production.^116,117^ Additionally, dietary phenolics are transformed into bioactive metabolites such as equol from isoflavonoids. Among cows fed oilseed crop concentration, soymeal supplementation yielded 116% higher levels of 3 measured phenolics (0.01 μg/mL) when compared with rapeseed meal (0.05 μg/mL).^76^ The elevated equol levels observed in milk from soymeal-fed cows or those fed clover in other studies discussed above highlight the capacity of rumen microbes to convert isoflavonoids into equol. This is particularly noteworthy given that equol is widely recognized for its potential human health-promoting properties, but is only produced by 30–50% of the population,^118^ indicating that milk could be a worthwhile dietary source for those who are unable to effectively metabolize equol from isoflavones.

When testing commercial milk samples from different farming systems, organic farms—characterized by grazing during the growing season—indicated ∼569% higher phenolic levels in milk based on a two-compound panel, ∼270% higher levels based on a 12-compound panel, and ∼108% higher level based on a 10-compound panel, compared with milk from conventional farms that provided concentrate-based feed (Table S2). Additionally, biodynamic farm milk—typically characterized by grazing a diversity of plant mixtures—contained ∼357% higher phenolic levels than conventional milk and ∼62% higher levels than organic milk based on a 10-compound panel.^119^ The elevated phenolic levels in biodynamic and organic systems are likely attributable to grazing fresh pasture, as well as increased plant competition for resources due to biodiversity, both of which are potentially important factors determining the phenolic richness of milk.^2^

In summary, phenolic concentrations in cow’s milk are strongly influenced by the botanical composition and conservation method of the diet, as well as by farming system and seasonal factors. Absolute concentrations are highly dependent on the size and scope of the analytical panel employed; therefore, within-study contrasts are more reliable than cross-study comparisons. Collectively, the current evidence indicates that fresh, biodiverse pastures—particularly those containing legumes such as red clover and birdsfoot trefoil—tend to yield the highest milk phenolic levels, followed by hay, with silage generally resulting in lower concentrations due to conservation-related degradation of phenolics.

#### 3.6.2 Goat Milk

Four studies reported TPC in goat milk with values ranging from 30 to 1390 μg GAE/mL (Figure 2).^49,61,64,66^ One study from Mexico reported 35% higher phenolics in milk from rangeland-grazing goats (88 μg GAE/mL) compared with animals fed alfalfa hay and corn silage (65 μg GAE/mL).^61^ In addition, they reported that unpasteurized milk (89 μg GAE/mL) had 39% higher phenolic levels than pasteurized milk (64 μg GAE/mL), and dry-season milk had 32% higher levels than rainy-season milk.^49^ The same group also reported that the phenolic content of pasture-fed goat cheese was ∼160% higher in raw milk cheese (780 mg/kg cheese) compared with cheese made from pasteurized milk (300 mg/kg).^120^ Seasonality can also affect the phenolic content of goat milk. For example, a study from Greece reported that milk phenolics were 26% higher in September (1390 μg GAE/mL) compared with March and June (1100 μg GAE/mL), 17% higher than May (1150 μg GAE/mL), 16% higher than April (1200 μg GAE/mL), 18% higher than July (1180 μg GAE/mL), and 11% higher than August (1240 μg GAE/mL) in goats grazing local grasslands.^43^

Furthermore, an Italian study reported that supplementing a beet pulp-based diet with grape Pomace increased phenolics 27% (38 μg GAE/mL) compared with the control (beet pulp-based diet), which was fed regular concentrate (30 μg GAE/mL).^64^ And 10% olive leaves increased TPC 33% (39.53 μg GAE/mL) compared with the control (29.75 μg GAE/mL).^63^ Altogether, studies in goat’s milk, consistent with findings in cow’s milk, indicate that fresh forages yield higher phenolic concentrations in milk compared with conserved forages and that phenolics are heat-sensitive compounds that deteriorate during pasteurization. In addition, dry-season plants—such as those occurring in September in Mediterranean environments—tend to contain higher phenolic levels, likely due to abiotic stress conditions that enhance phenolic synthesis in plants, thereby improve the phenolic quality of forage.

In addition to TPC assays in goat’s milk, targeted phenolic analyses reported concentrations ranging from ∼10 to 26.79 μg/mL across two studies (Figure 3). One study, utilizing a panel of 25 compounds, found that phenolics in semiskimmed UHT milk were 77% higher than in whole UHT milk purchased from the market, whereas a study using a panel of five compounds measured a concentration of 0.01 μg/mL in commercial samples from Hungary.^108^ The higher phenolic levels observed in semiskimmed UHT milk relative to whole UHT milk may, in part, reflect concentration effects, since these compounds are primarily water-soluble and semiskimmed milk contains less fat. Altogether, the two studies highlight the substantial influence of analytical panel selection on reported concentrations, while differences may also reflect variations in animal feeding practices. Ianni et al.,^63^ measured 19 phenolics in the milk of goats fed 10% olive leaves, with total concentration reaching 0.45 μg/mL.

#### 3.6.3 Sheep Milk

Two studies, both from Italy, reported TP levels ranging from 33 to 57 μg GAE/mL in sheep’s milk (Figure 2). A study comparing Sulla forage to barley meal showed that Sulla fresh forage yielded 15% higher TPC levels (58 μg GAE/mL) than barley meal (49 μg GAE/mL), while the Sulla fresh forage + barley meal diet yielded 56.2 μg GAE/mL, essentially similar to Sulla fresh forage feeding.^62^ In a separate trial, supplementing beet pulp with grape pomace increased phenolics by 15% (38 μg GAE/mL) compared with a beet-pulp diet control (33 μg GAE/mL).^64^ Altogether, these results indicate that fresh forage and grape pomace supplementation enhances sheep-milk phenolics relative to cereal-based or beet-pulp diets. These findings align with the literature on cow and goat milk, described above. In particular, fresh forages are a primary source of phenolics and generally yield milk with higher levels than animals fed conserved feeds and/or cereal or fibrous byproducts (e.g., barley meal, beet pulp), while phenolic-rich feed byproducts such as grape pomace can augment phenolic intake.

Only one study in this review applied targeted phenolic analysis to sheep milk (Figure 3). Using a panel of 21 compounds, the study reported that milk from ewes fed grape pomace contained 41% higher phenolic levels (1.28 μg/mL) than milk from ewes fed beet pulp (1.28 μg/mL).^54^ Compared with the ∼15% difference observed for TPC, the targeted assay showed a more pronounced effect, underscoring the limitations of comparing targeted-panel data with total phenolics. Moreover, only four of the 21 compounds—rosmarinic acid, epigallocatechin gallate, kaempferol, and luteolin—accounted for most of the difference in the grape-pomace group, whereas beet pulp is richer in betalains, which were not included in the analytical panel. Cross-study comparisons should therefore be made cautiously, and future work should pair TPC with standardized, broader (or untargeted) profiling to capture the full phenolic spectrum, while others have made the case to drop crude assays like TPC altogether and to focus on more sophisticated chromatographic techniques instead when profiling phenolics.^68^

### 3.7 Phenolics Concentration in Ruminant Meat

Of the 39 articles screened, four reported quantitative data on phenolics in ruminant meat (Table S2). One Italian study reported TPC in sheep meat, while a UK study quantified five phenolic compounds in sheep meat. In beef cattle, two studies reported phenolic concentrations with a UK study using a panel of five compounds and a US study using a panel of ten compounds. Given the limited quantitative evidence, we synthesized results across species.

In sheep meat, TPC (expressed as μg GAEs per g; μg GAE/g) under ad libitum feeding was 0.19 μg GAE/g in the control group.^62^ Supplementation with 20% wheat bran decreased TPC by ∼11% relative to this control (to ∼0.17 μg of GAE/g). Under restricted feeding conditions, the control was 0.29 μg of GAE/g, while adding 20% wheat bran increased TPC by ∼47% versus the control (to 0.42 μg of GAE/g). Notably, the restricted-feeding control was ∼53% higher than the ad libitum control, indicating a substantial effect of feeding regime independent of supplementation. Sheep fed 20% wheat bran (with alfalfa pelleted hay, fava beans, and barley grains) exhibited the highest TPC observed (0.42 μg GAE/g) among all meat samples included in this review. Additionally, a targeted phenolic analysis (panel of five compounds) reported concentrations of 0.06 μg/g across the five compounds in a commercial sheep meat sample.^108^ These findings suggest that both the feeding regime (e.g., restricted vs ad libitum) and diet composition (e.g., wheat bran inclusion) can significantly influence phenolic content in sheep meat.

One commercial sample analyzed with a five-compound panel reported levels of 0.15 μg/g in cattle meat,^108^ while another study using a ten-compound panel found that meat from cattle finished on diverse pasture had phenolics that were ∼39% higher (217 μg/g) than total mixed ration feeding (TMR; 156 μg/g) and ∼37% higher than TMR + 5% grapeseed extract (159 μg/g).^5^ Collectively, the limited sheep and cattle data sets indicate that meat phenolic concentrations can vary substantially with feeding regimen/diet. However, cross-study comparisons are constrained by small sample sizes, heterogeneous analytical panels (TPC vs targeted compounds), differing units (μg GAE/g vs μg/g), and potential under-representation of key phenolic classes.

### 3.8 Phenolic Diversity in Ruminant Milk and Meat

Besides total content, diversity of phenolics is likely to be important, as different compounds exhibit different metabolic effects. In total, 356 phenolic compounds were identified across 29 studies on milk, of which 193 were unique to milk and not reported in meat (Table 1). Sheep milk exhibited the greatest diversity, with 160 compounds, of which 110 were unique (six articles: refs 54, 56, 63, 84, 121, and 122). This was followed by cow milk with 98 compounds, of which 54 were unique (16 articles: refs 19, 49, 73–76 ,^108–110^ ,^113^,^114^,^119^, and^123–126^ ), goat milk with 70 compounds, of which 29 were unique (four articles: refs 49, 122, 127, and 128), and buffalo milk with 28 nonunique compounds (one article: ref 122). Class-level patterns are presented in Figure 4. Across all species, phenolic acids and flavonoids dominated the individual compounds found in the milk of sheep (50 phenolics; 49 flavonoids), cows (27 phenolics; 19 flavonoids), goats (25 phenolics; 21 flavonoids), and buffalo (13 phenolics; one flavonoid), respectively. This was followed by polyphenols (19, 14, 12, and 9 compounds for sheep, goat, cow, and buffalo, respectively) and other organic compounds (13, 7, 12, and 2 compounds for sheep, goat, cow, and buffalo, respectively). Lignans and quinones were only reported in the milk of cow, goat, and sheep, containing five, three, and seven lignans; and one, five, and four quinones, respectively. Stilbenes were reported only in sheep milk (four compounds), and tannins were found only in sheep milk (three compounds) and goat milk (one compound). Based on the current literature, sheep milk exhibits the most diverse phenolic profile, encompassing all ten distinct classes, followed by goat milk with nine classes, cow milk with eight, and buffalo milk with six; however, this can also reflect study bias, with only one investigating buffalo milk. Across studies profiling meat included in this review, we identified 203 phenolic compounds (Table 1). Across seven articles, four focused on beef cattle,^4,5,12,57^ and one each examined goat,^80^ sheep,^108^ and bison.^6^ Beef exhibited the greatest diversity with 164 compounds, and 132 were only reported in this meat, followed by 17 compounds in goat (all unique), bison with 12, and sheep with 5 compounds. At the class level, beef was reported to contain 39 organic compounds, 39 flavonoids, 32 alkaloids, 16 phenolic acids, 15 polyphenols, nine coumarins, two quinones, and one compound each from the stilbene and tannin categories. Studies on sheep meat reported five flavonoids, while studies in goats meat identified three additional flavonoids, five phenolic acids, two other organic compounds, and one compound each from the stilbene and lignan classes. A bison study reported five polyphenols, four phenolic acids, and three other organic compounds. Collectively, sheep milk currently exhibits the broadest class diversity, while beef shows the most unique phenolics of all studied meats; however, the pattern in beef is almost certainly limited by the available data, as more studies have profiled beef than other meats. Therefore, the research focus needs to be increased beyond beef cattle.

**Figure 4:**
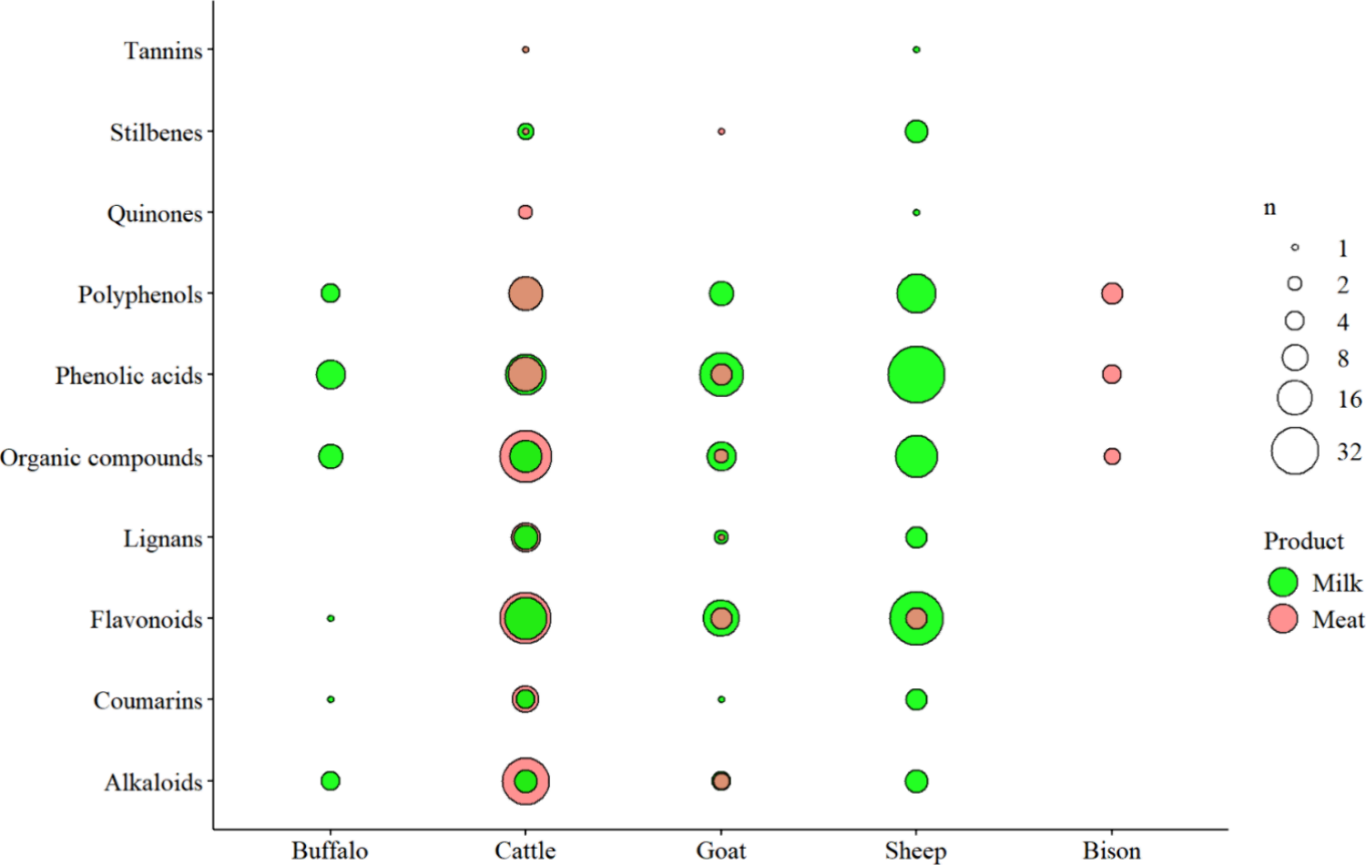
Phenolic diversity across animal-source foods identified by the current systematic review (*n* = 39 studies). Circle size is the number of phenolics reported within each class; circle color indicates the food product. Because flavonoids, phenolic acids, and other polyphenols vary widely, these classes are presented separately.

**Table 1:** Summary of 29 Studies Report Phenolics in Meat and/or Milk of Selected Animals in This Systematic Review with Total Number Compounds, Unique Compounds, and Total Citations for Each Product^a^

latin name/species	common name	product	no. of citations	total phenolics	unique phenolics
*Bos taurus*	cattle	milk	17	98	54
		meat	4	164	132
*Capra aegagrus hircus*	goat	milk	5	70	29
		meat	1	17	17
*Ovis aries*	sheep	milk	6	160	110
		meat	1	5	0
*Bubalus bubalis*	buffalo	milk	1	28	0
*Bison*	bison	meat	1	12	0

a Values are counts of phenolics summarized across 39 studies, reported as Total (all detections) and Unique (distinct compounds after deduplication with any other product in this review).

### 3.9 Medicinal-Plant Phenolics in the Ruminant Milk and Meat

Beyond their capacity to accumulate a wide array of phenolics, ruminant meat and milk also introduce phenolics from medicinal or nonstaple plants into the human food chain. In our review, we identified 18 such compounds (Table 2). For instance, aloperine was reported at 167% higher levels in ground beef compared with plant-based meat, which is a compound derived from the necklacepod (*Sophora alopecuroides*) and historically studied for its anti-inflammatory and anticancer activity.^129^ Brucine, also reported in ground beef, is likely derived from *Strychnos nux-vomica* and has known anti-inflammatory/analgesic and antiulcer properties.^130–133^ Additionally, Corydaline from *Corydalis yanhusuo*, a plant used since the Tang period (seventh–10th centuries AD) for pain relief, which is now linked to analgesic and anti-inflammatory mechanisms,^134–137^ was also found in beef.^129^ Dictamnine, derived from *Dictamnus dasycarpus*, with antibacterial, antiallergic, and anticancer effects,^138–141^ and dihydropalmatine (also from *Corydalis yanhusuo*) are also reported in beef. Dihydropalmatine shows dopaminergic modulation (including D2-related effects in migraine models) and anti-inflammatory activity, and has been tested against P388/L1210 leukemia.^142–145^ Additionally, Pongamol from *Pongamia pinnata* demonstrates antidiabetic potential via enhanced GLUT4 translocation and cellular glucose uptake;^146,147^ Gomisin m2, annotated in cow milk, from *Schisandra rubriflora* has shown in vitro antiviral activity and antiallergic properties;^148,149^ Salicin, detected in the milk of goat fed silage from *Salix alba*, has reported neuro- and vascular protective properties;^150–152^ while Schisandrin, annotated in sheep milk and derived from *Schisandra chinensis*, has potential cardioprotective, anticancer, and immunomodulatory properties.^153–155^ Collectively, these compounds potentially act first in the animal—supporting anti-inflammatory, antioxidant, and metabolic defenses—with a certain amount then accumulating in tissues or being transferred into milk, thereby providing low-dose human exposure. Whether this confers human health benefits remains to be studied. Additionally, it is important to highlight that all the medicinal-plant phenolics were reported in untargeted metabolomics studies, thereby limiting quantitative outcomes.

**Table 2:**
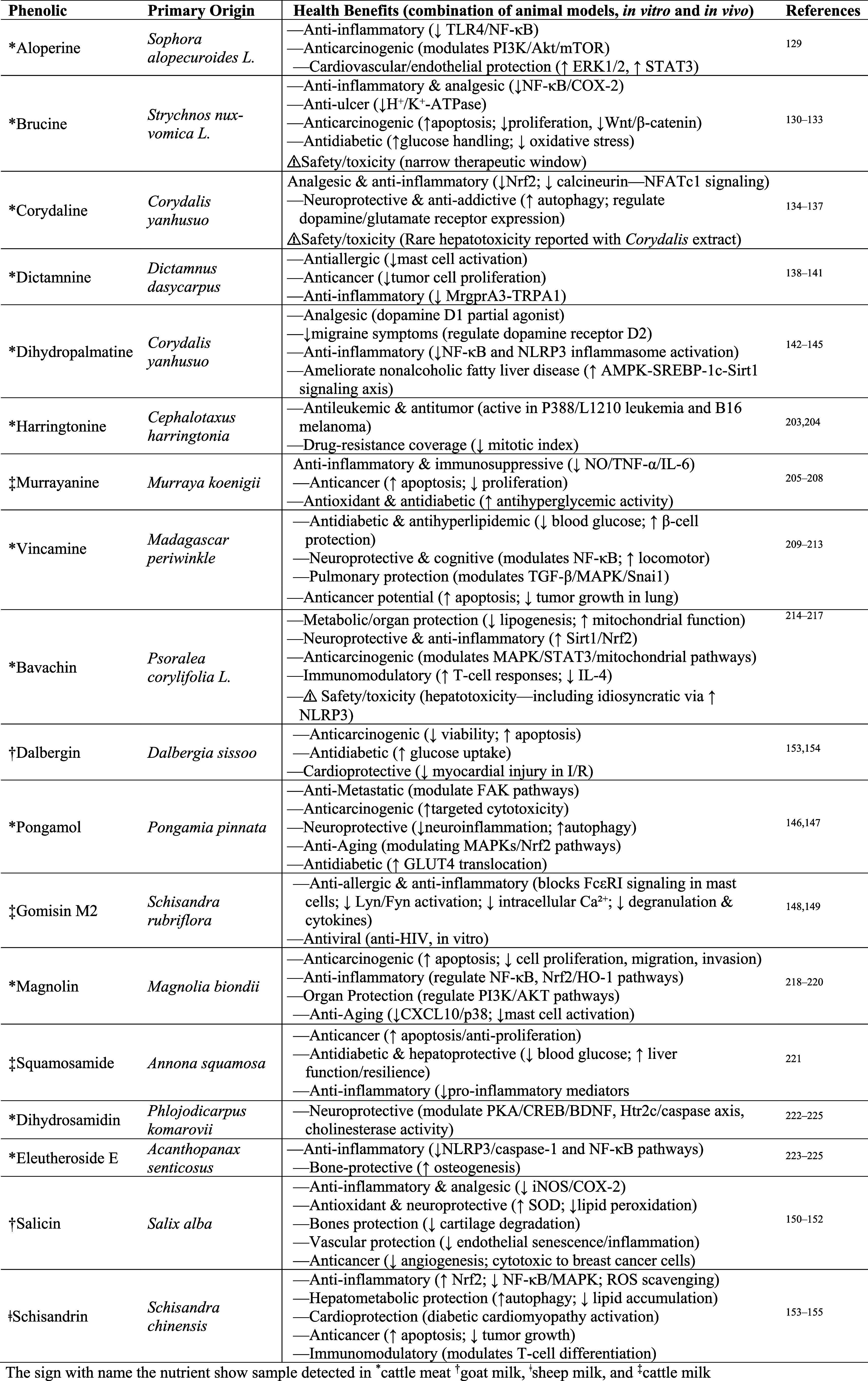
Therapeutic Potential of Medicinal Plants Phenolics Identified in the Meta-Analysis in the Ruminant’s Meat and Milk

It is also important to note that certain phenolic compounds, such as brucine, Corydaline, and bavachin, possess narrow therapeutic windows and can exert toxic effects at higher levels of intake. For example, studies in mice have shown that bavachin can induce mild hepatic steatosis at doses of approximately 97 mg/kg.^156^ By extrapolation, a concentration of ∼1 mg/g of bavachin in meat (based on a 150 g beef serving, an average 1.5 kg human liver, and assuming 100% absorption) could theoretically approach hepatotoxic levels in humans. However, it is highly unlikely for animal tissues to accumulate such concentrations without adverse effects on the animals themselves, especially given that the maximum TPC reported in this review was only 1.62 mg of GAE/g of meat.

Grazing animals often exhibit improved mitochondrial function, more robust immune responses, and lower stress (e.g., reduced cortisol) compared with animals in confinement, which potentially relates, in part, to the presence of these compounds in their biological systems.^4,6,157,158^ It has been postulated that grazing on diverse pastures promotes a flavor–feedback mechanism,^159^ whereby animals associate the taste of plants with their postingestive consequences.^160^ This mechanism allows them to select portions from different plants, including medicinal species, to balance nutrient intake and self-medicate.^161,162^ For example, lambs developed a preference for low-quality flavored foods when paired with intraruminal infusions of energy (starch) or protein (casein), and grain-fed lambs showed a preference for sodium bicarbonate to counteract rumen acidosis.^163^ In short, ruminant flavor–feedback mechanisms likely facilitate the inclusion of medicinal plants in their diets, thereby supporting animal well-being,^164^ while introducing trace levels of bioactive compounds into the human food chain through milk and meat.^165^ Although trophic reduction of these molecules may lessen their potency, it simultaneously reduces toxicity risks (e.g., from brucine) while enabling long-term,^166,167^ low-level exposure that could hold subtle physiological relevance.^166,168^

### 3.10 Bioactivity of Phenolic-Derived Mammalian Metabolites in Their Meat and Milk

Considering that <5% of ingested polyphenols appear in the plasma as parent forms, the major circulating compounds are host–microbial metabolites.^101^ Building on this metabolite-centric view, we summarize the biological significance of the metabolites that appeared most frequently in our search in Table 3. Hippuric acid is one such compound, which ranged 0.01–0.07 μg/mL in milk and 0.05–0.13 μg/g in meat (Figure S3a). Hippuric acid is a gut-microbial metabolite produced from polyphenols, which has been linked to improved fasting glucose and insulin secretion, and to reduced colitis in animal models; indirectly, it also may indicate healthy aging.^169–174^ Additionally, hippuric acid is associated with improved gut microbial diversity and decreased risk of metabolic syndrome in humans without renal dysfunction.^175,176^ P-Cresol sulfate, another polyphenol-derived microbial metabolite, has been shown to have anti-inflammatory properties in primary biliary cholangitis and lung cells^177–179^ and is also commonly reported in studies comparing grass-fed vs grain-fed livestock.^4–6^ Enterolactone, ranging from 0.01 to 0.23 μg/mL in milk (Figure S3b), has been associated with a lower risk of hormone-dependent cancers, reduced proliferation of estrogen-sensitive cells, and improved cardiometabolic health.^180–183^ Equol, particularly in its sulfate form, exhibits high affinity for estrogen receptor (ER)-β and has been linked to improvements in menopausal symptoms and bone health, with antioxidant and anti-inflammatory properties in preclinical models.^184–187^ Pyrogallol (and catechol) sulfates, also commonly reported metabolites in meat and milk, have been reported to possess antioxidant, anti-inflammatory, and anticarcinogenic properties, and to modulate carbohydrate metabolism.^188–192^ Additionally, recent in vivo work indicates that goat’s milk enriched in phenolic compounds—at a dose equivalent to the daily human intake of 250 mL of fresh goat’s milk for a 60 kg adult—decreased body weight and fat mass, improved glucose tolerance, and prevented adipose tissue hypertrophy and hepatic steatosis in mice fed a high-fat diet. Another study in humans found that daily consumption of pecorino cheese—made from sheep foraged on diverse pastures—for 10 weeks reduced circulating levels of pro-inflammatory cytokines and improved erythrocyte deformability.^176^ Overall, initial evidence supports the notion of preformed phenolic metabolites from meat and milk as plausible mediators of antioxidant, anti-inflammatory, cardiometabolic, and neuroprotective effects; however, more studies in vivo in humans are necessary to confirm their efficacy.

**Table 3:**
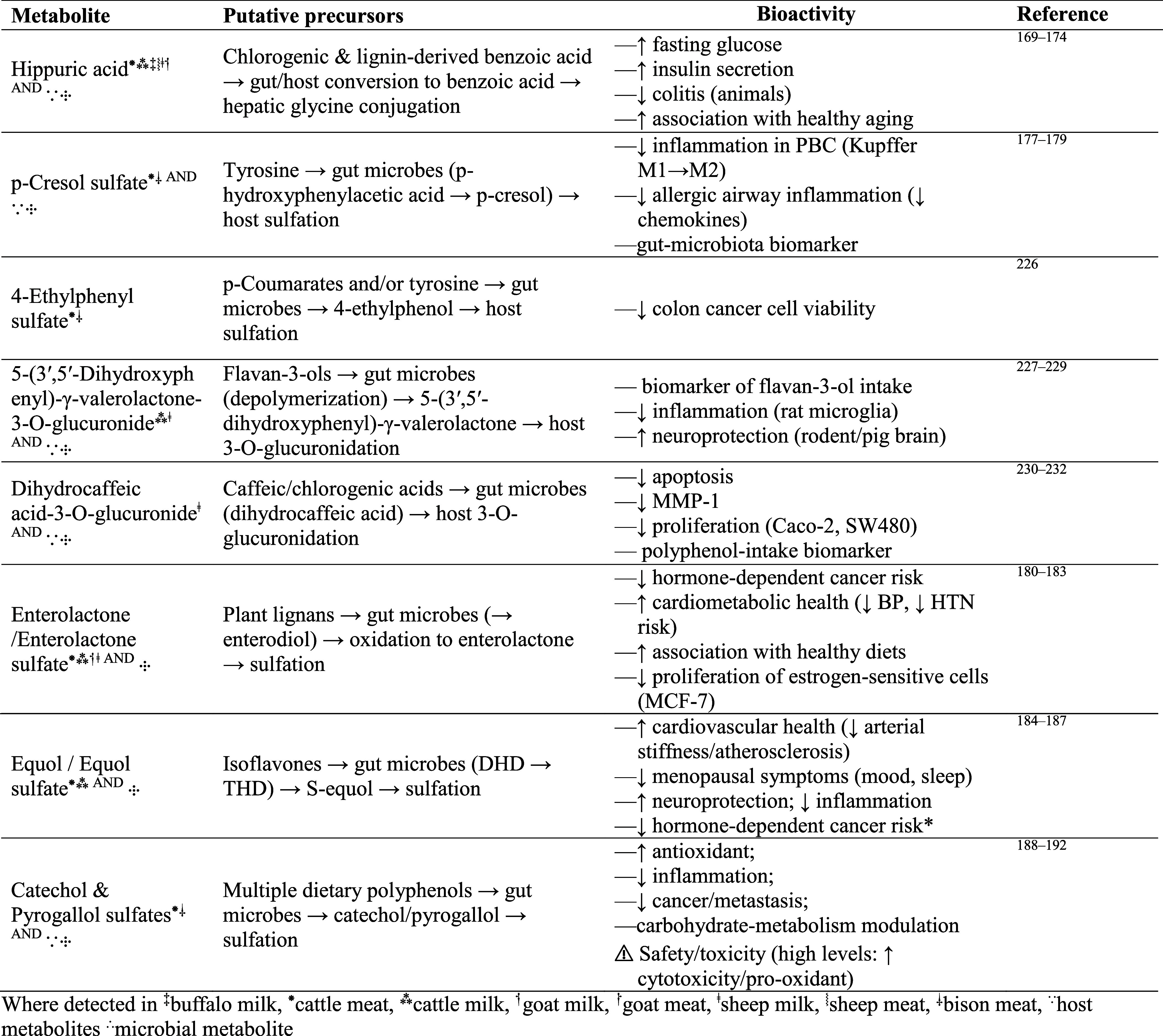
Phenolic-Derived Mammalian Metabolites Identified in the Meta-Analysis: Putative Precursors and Their Health Effects

It is important to highlight that the above-mentioned phenolic-derived metabolites are also commonly reported in the human metabolome, as humans, being mammals as well, extensively metabolize phenolics through gut microbial breakdown and liver conjugation. Therefore, exposure to premetabolized phenolics through meat and milk may reduce the metabolic cost for consumers, since the gut–liver axis will need to exert less enzymatic activity to convert these compounds into smaller, more water-soluble forms.^193–195^ This may reduce oxidative load and help overcome microbiome limitations (e.g., nonequol producers). Metabolites from meat and milk should arguably be viewed as supplementary to direct exposure from plant-based foods in most cases, but they can also act alone in some cases (e.g., with a limited gut microbiome) as low-dose signaling molecules at target tissues.^196,197^ Considering the trophic dilution along the pasture → animal → food chain, exposures likely stay within physiological, nontoxic ranges.^198,199^ Additionally, as discussed above, forages consumed by ruminants may also introduce various unique phenolic metabolites into the human diet from plants otherwise not consumed.

Western diets typically provide 600–1,000 mg GAE/day of phenolics, mainly from coffee, tea, fruits, and vegetables.^200–202^ Based on the highest values in our data set, one serving (240 mL) of goat milk provides ∼334 mg GAE (1,390 μg GAE/mL; September milk, Greece) while cow milk provides up to ∼6 to 14 mg GAE per 240 mL serving (Figure 2). These servings correspond to ∼33 to 56% (goat milk) and ∼2% (cow milk), of typical Western daily intake. On a food basis comparison, red wine contains ∼340 mg GAE per 150 mL (reported range: ∼240 to 520 mg/150 mL),^44,45^ thus goat andcow milk can deliver ∼98 and ∼1 to 2% of the phenolics in a glass of red wine, respectively. A 240 mL serving of green tea typically supplies up to 320 mg GAE and relative to this, goat and cow milk provide ∼104, and ∼2 to 9, respectively.^44–46^ Since small-ruminant milks can supply up to 104% GAE of well-known phenolic-rich foods, it can be speculated that milk from some species may make a materially meaningful contribution to total dietary polyphenol exposure in the Western diet, and its role should not be underestimated, particularly when animals are grazing diverse pasture and/or are consuming phenolic-rich feed. However, phenolics from meat and milk should not be viewed as replacements for direct plant food consumption in the human diet, in our view, given that plants introduce a plethora of phenolics not found in animal-sourced foods and often in higher concentrations. Nonetheless, animal-sourced foods can provide metabolized versions of phenolics in addition to health-promoting compounds otherwise not readily obtained in human health.^203–232^

### 3.11 Challenges and Limitations in Current Research

The primary challenge in evaluating ruminants’ trophic-level contribution of phenolics to the food chain is the limited characterization of downstream metabolism in the rumen and gut microbiota, intestinal enterocytes, liver, and kidneys. These biotransformation pathways are likely to generate a myriad of metabolites, as indicated by tracer work, as previous work indicates that <5% of phenolics in the serum appear as their parent plant compounds but their metabolites reach a higher peak concentration after intake.^103^ Thus, the trace parent phenolics in meat and milk also reflect extensive microbial/host metabolizing capacity rather than lower exposure. Because many metabolites exhibit biological activities distinct from their precursors (e.g., equol versus its isoflavone parents), comparing TPC or parent phenolics in such matrices with those found in plants may introduce source-attribution bias between plant and animal sources.

Detecting these compounds in animal matrices is challenging because they occur in relatively trace levels compared with proteins and lipids. The purification process (e.g., defatting, protein precipitation, solid phase extraction, and/or filtration) often leads to losses; however, inadequate purification causes severe ion suppression (from phospholipids, salts, and peptides) in MS assays, thereby reducing sensitivity. Moreover, many “discovery” metabolomics workflows report phenolics only as a small subset of a global profile and employ extraction, chromatography, and annotation protocols that are optimized for central metabolites, which potentially overlook phenolic abundance.

Advanced analytical techniques, such as QTOF and Orbitrap, offer high sensitivity and mass accuracy, enabling better detection of trace levels of bioactive compounds. These capabilities surpass those of traditional UV–vis or PDA detectors. However, the effectiveness of QTOF-MS and Orbitrap-MS is constrained by the availability and comprehensiveness of spectral libraries for identifying phenolic compounds, particularly those that have been less studied or novel. Targeted analytical methods face additional challenges due to the limited availability of pure analytical standards, especially for conjugated forms of phenolics, such as sulfates, glucuronides, and glycosides, which are the predominant forms in animal-sourced foods. The scarcity of these standards hampers the quantification of downstream metabolites of parent compounds found in plants, which are often critical for understanding the biological activity and bioavailability of phenolics. This limitation affects the reliability and reproducibility of the results, particularly in studies aiming to quantify specific phenolic metabolites.

Despite these limitations, emerging evidence indicates that when ruminants graze on biodiverse pastures, their meat and milk contain a richer and more diverse array of phenolics compared with animals grazing monocultures or fed grain-based concentrates. Additionally, seasonality, pasture management, and feed sources can also impact the phenolic contents of meat and milk. Emerging evidence suggests that, in some contexts, milk—especially from small ruminants such as goats and sheep—may contribute meaningfully to daily polyphenol intake, complementing plant foods (e.g., a serving of goat milk can provide up to 104% of the GAE phenolics found in a serving of green tea). However, it is important to note that phenolic metabolites from animal-sourced foods should be viewed as complementary rather than as substitutes for direct consumption of plants due to the differences in metabolomes.

Importantly, ruminants can “upcycle” plant phenolics into mammalian metabolites (e.g., hippuric acid, equol, and enterolactone), which likely represent the predominant forms of plant parent compounds entering human circulation. Additionally, ruminants consume plants with potential medicinal value that are otherwise not consumed by humans. These metabolites have been linked to reduced inflammation and oxidative stress, highlighting potential cardiometabolic benefits when consumed within healthy dietary patterns.

To conclude, ruminants act as biological mediators in the soil–plant–animal–human continuum, extending the diversity and magnitude of phenolic metabolites available in human diets. Advancing this field will require interdisciplinary collaboration across agriculture, ecology, nutrition, analytical chemistry, and data science, with an emphasis on standardized sampling, improved extraction methods, and expanded spectral libraries. Such integration can move discussions of meat and milk beyond reductionism toward a fuller understanding of how phytochemical-rich grazing systems and feed sources can simultaneously sustain ecosystems, improve animal well-being, and enhance human health.

## Supplementary Material



## Notes

Figure S1. PRISMA (Preferred Reporting Items for Systematic Reviews and Meta-Analyses) flow diagram summarizing the literature search and study selection process followed to selected eligible studied to be this systematically review. Figure S2. Risk of bias and quality of reporting assessment based on SYRCLE’s risk of bias tool 1: (A) indicator of quality of reporting included study design and objectives, population and sample characteristics, intervention and comparison groups, analytical methods, data analysis and presentation, ethical considerations, funding and conflicts of interest (B) risk-of-bias analysis in selection, measurement, confounding, and selective reporting. Table S1 List of phenolic compounds identified in selected ruminants meat and milk samples from studies included in the systematic review, organized by matrix (species/product). Table S2. Study-level data extraction for 39 studies, including sample type; country of origin; experimental group (e.g., breed, fresh vs conserved forage, short- vs long-term pasture, month of year, commercial samples, milk-fat content); measured phenolics (relevant compounds extracted from metabolomics data sets); concentrations (where reported, with n per group); analytical platform; and references (PDF)

## Abbreviations Used

3-PPA: 3-phenylpropionic acid
COMT: catechol-O-methyltransferase
CTs: condensed tannins
DOAJ: Directory of Open Access Journals
ER-β: estrogen receptor beta
FTIR: Fourier-transform infrared spectroscopy
GAE: gallic acid equivalents
GC: gas chromatography
GC-MS/MS: gas chromatography–tandem mass spectrometry
HPLC: high-performance liquid chromatography
HRAM: high-resolution accurate mass
HIV: human immunodeficiency virus (appears in “anti-HIV”)
HP-2: (enzyme prep type) *Helix pomatia* β-glucuronidase/sulfatase “HP-2
LC: liquid chromatography
LC-MS/MS: liquid chromatography–tandem mass spectrometry
*m*/*z*: mass-to-charge ratio
MS: mass spectrometry
MS/MS: tandem mass spectrometry
PDA: photodiode array (detector)
PRISMA: Preferred Reporting Items for Systematic Reviews and Meta-Analyses
QqQ: triple quadrupole (mass spectrometer)
QTOF: quadrupole time-of-flight
QTOF-MS: quadrupole time-of-flight mass spectrometry
SGLT1: sodium-dependent glucose transporter 1
SULT(s): sulfotransferase(s)
SYRCLE: Systematic Review Centre for Laboratory animal Experimentation
TMR: total mixed ration
TPC: total phenolic content
UGT(s): UDP-glucuronosyltransferase(s)
UHT: ultrahigh-temperature (processing)
UK: United Kingdom
US/USA: United States/United States of America
UV–Vis: ultraviolet–visible (spectrophotometry)
v/v: volume/volume (solution ratio)
