# Supplementary material for: Phenolic Compounds and Derivatives in Ruminant Meat and Milk: A Systematic Review
Source: J Agric Food Chem. 2025 Nov 14;73(47):29961–82. doi: 10.1021/acs.jafc.5c06118 (PMC12679856; doi:10.1021/acs.jafc.5c06118)
Supplement: Supplementary file 1 [file jf5c06118_si_001.pdf]

Supplementary Information

Phenolic Compounds and Derivatives in Ruminant Meat and Milk: A Systematic Review

Muhammad Ahsin[a]*, Sulaiman K. Matarneh[a], Kara J. Thornton[b], Scott Kronberg[c], Mamoona Amir[d], and Stephan van Vliet[a]*

[a]Department of Nutrition, Dietetics & Food Sciences, College of Agriculture and Applied Sciences, Utah State University, Logan, UT 84322, USA

[b]Department of Animal, Dairy, and Veterinary Sciences, Utah State University, Logan, UT 84322, USA

[c]USDA-Agricultural Research Service, Mandan, ND, USA 58554

[d]Bahauddin Zakariya University, Department of Animal Food Products Technology, Faculty of Food Science and Nutrition, Multan, Punjab, PK 60800

*Correspondence to: muhammad.ahsin@usu.edu; stephan.vanvliet@usu.edu

Supplementary Information

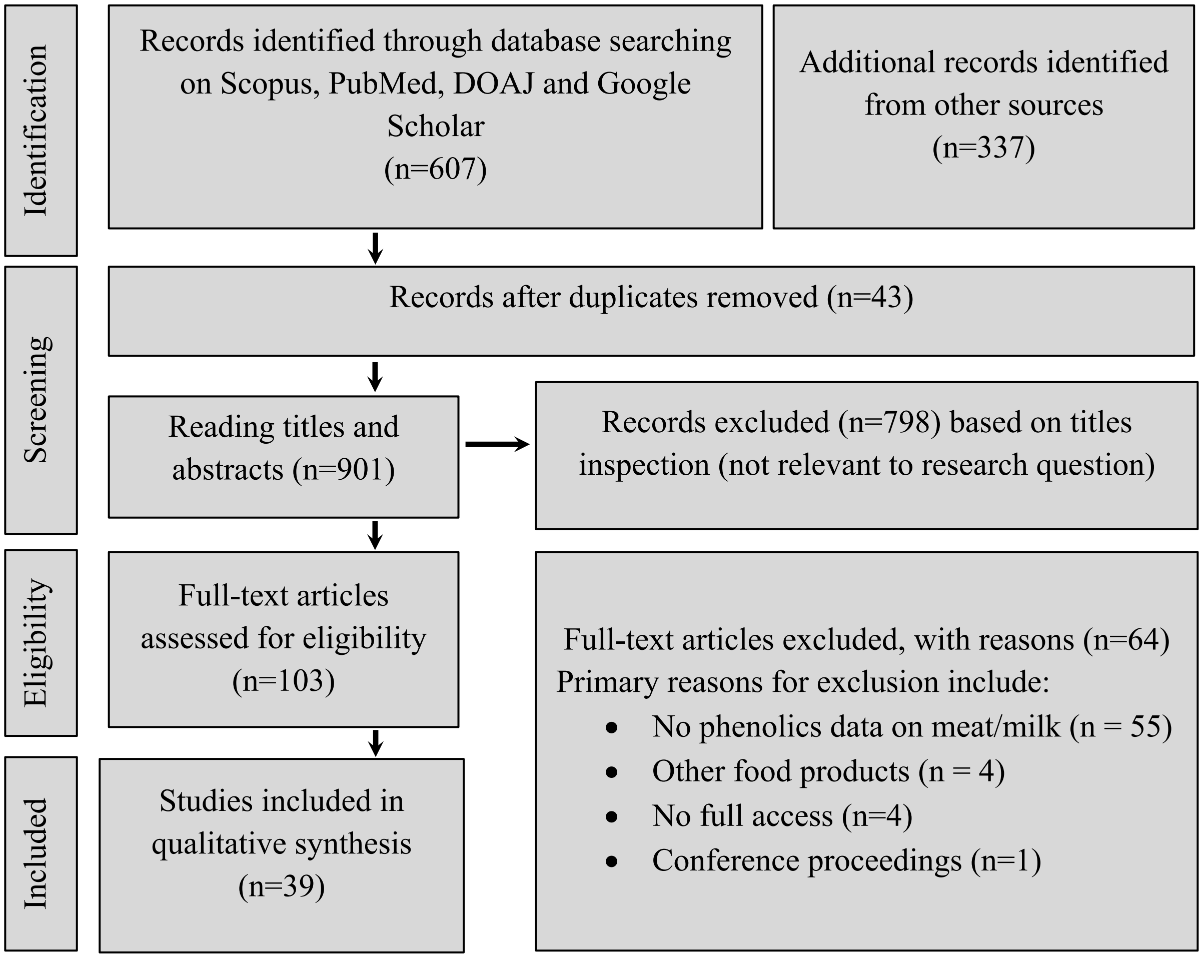

Supplemental Figure S1. PRISMA (Preferred Reporting Items for Systematic Reviews and Meta-Analyses) flow diagram summarizing the literature search and study selection process followed to selected eligible studied to be this systematically review

A

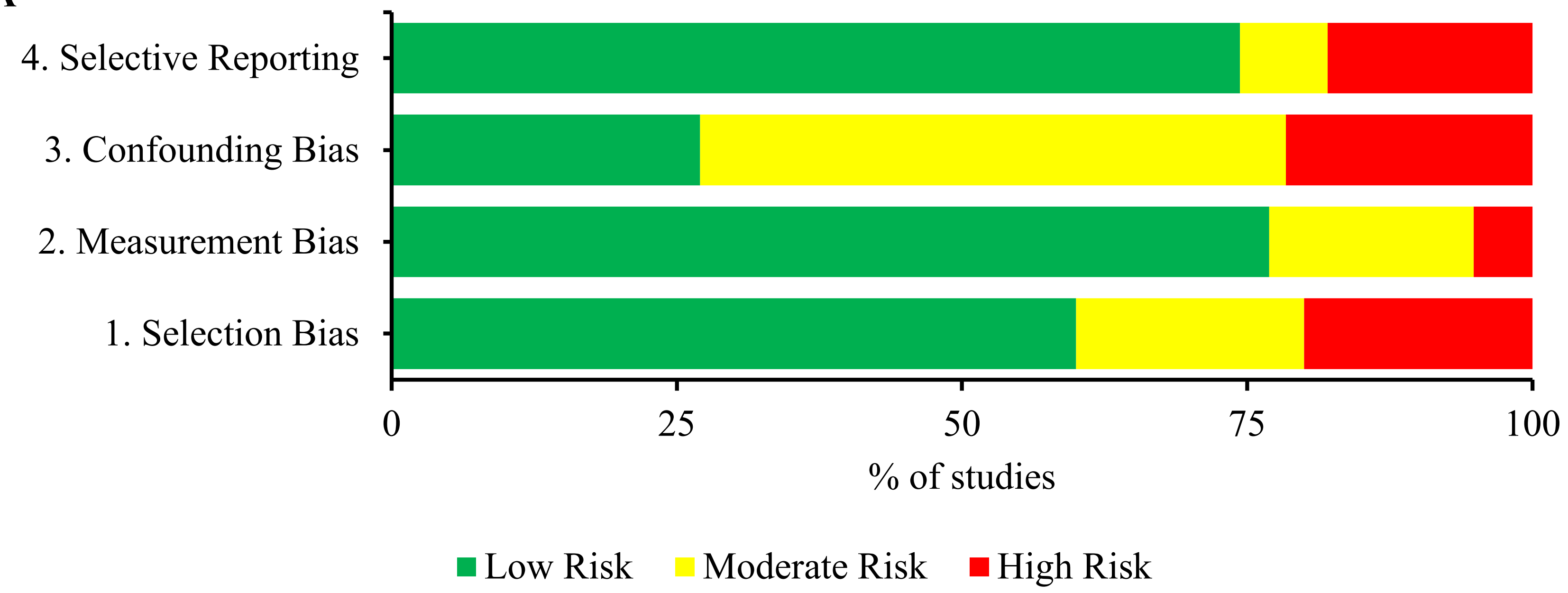

Supplemental Figure S2. Risk of bias and quality of reporting assessment based on syrcle's risk of bias tool [1]: (A) indicator of quality of reporting included study design and objectives, population and sample characteristics, intervention and comparison groups, analytical methods, data analysis and presentation, ethical considerations, funding and conflicts of interest (B) risk-of-bias analysis in selection, measurement, confounding, and selective reporting

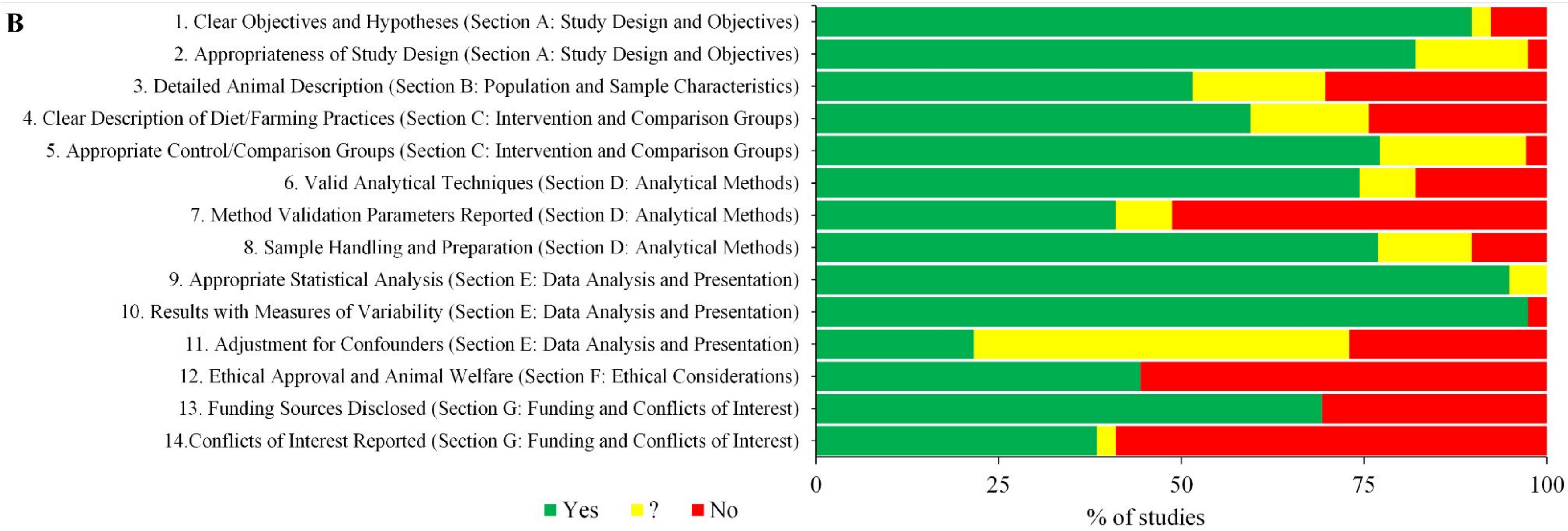

Supplemental Figure S2. Risk of bias and Quality of reporting assessment based on SYRCLE's risk of bias tool[1]: (A) Indicator of quality of reporting included study design and objectives, population and sample characteristics, intervention and comparison groups, analytical methods, data analysis and presentation, ethical considerations, funding and conflicts of interest (B) risk-of-bias analysis in selection, measurement, confounding, and selective reporting.

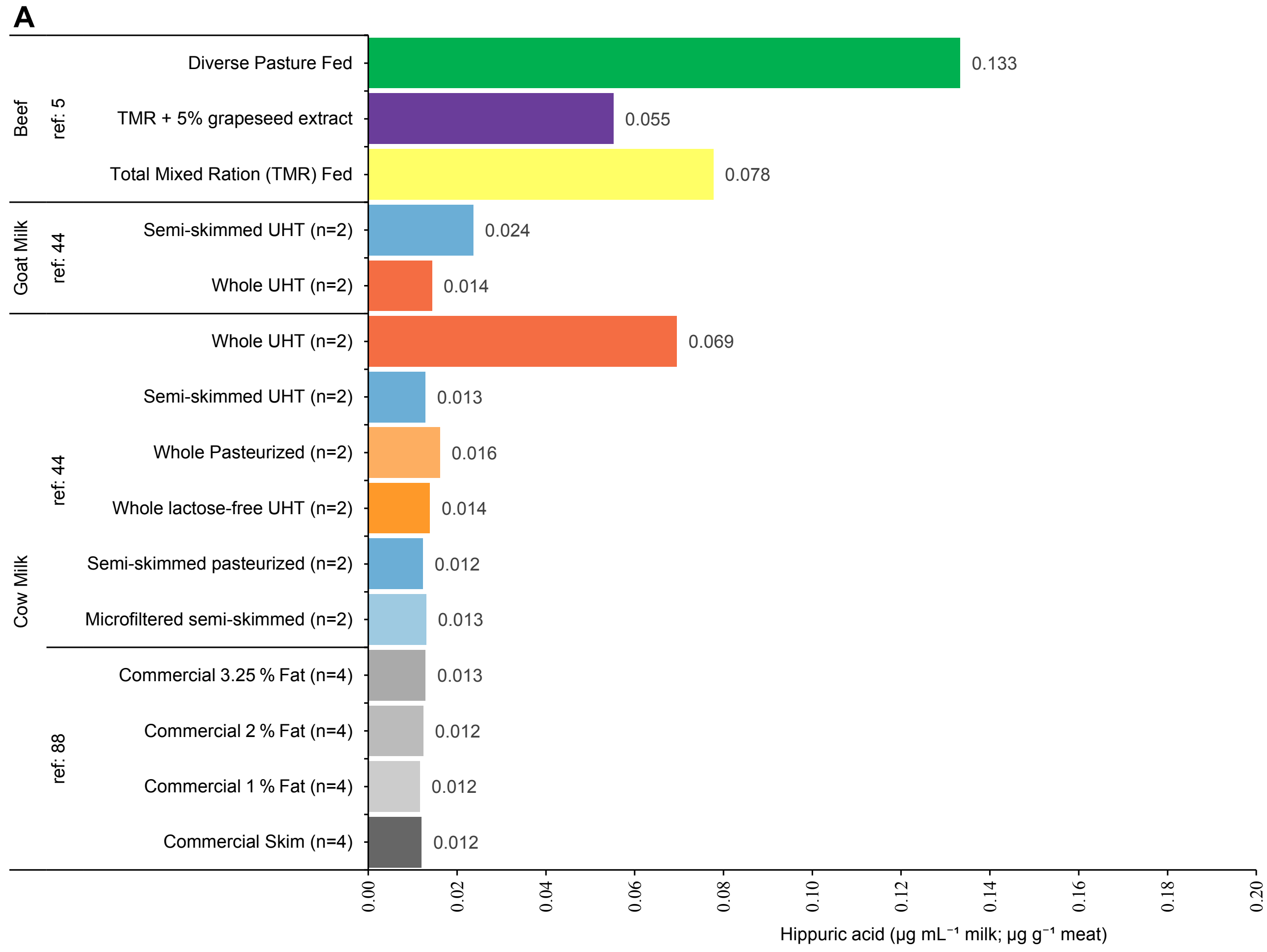

Supplemental Figure S3. Concentrations of individual phenolic compounds reported across different products and experimental groups, with corresponding references. (B) Hippuric acid.

B

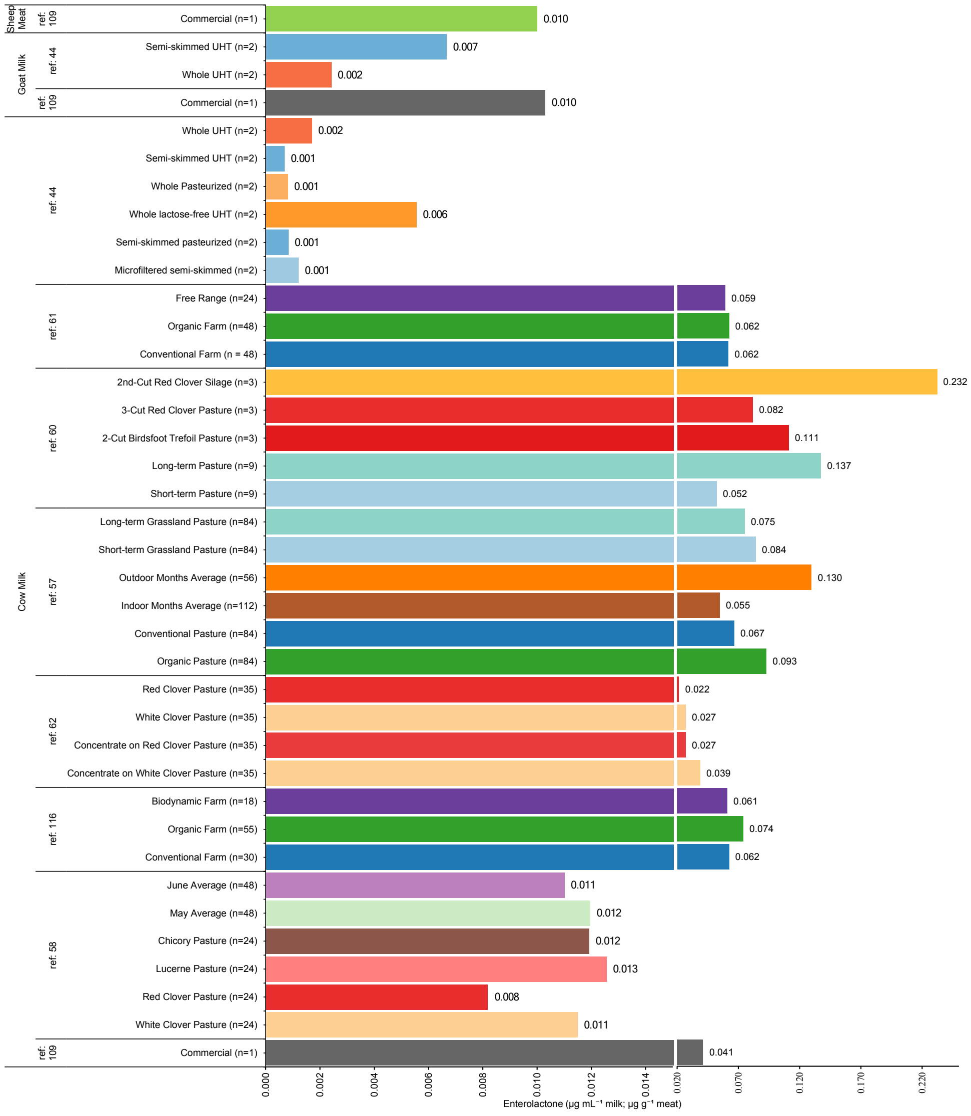

Supplemental Figure S3. Concentrations of individual phenolic compounds reported across different products and experimental groups, with corresponding references. (B) Enterolactone.

Supplementary Table S1. Supplementary Table S1 List of phenolic compounds identified in selected ruminants meat and milk samples from studies included in the systematic review, organized by matrix (species/product).

**Cattle meat**		
(R)-(-)-phenylephrine	Coumaric acid 4-o-glucoside	Magnoflorine
1-(2-hydroxy-3-methyl)-butyl-hydrocotarnine	Cyproconazole	Magnolin
1,2,4-trimethoxy-5-propenylbenzene	Daidzin 6'-o-malonate	Methyl 3-(4-hydroxy-6-methyl-2-oxo-2h-pyran-3-yl)-3-phenylpropanoate
1-methyl-2-undecylquinolin-4-one	Delphinidin	
2,3-dihydroxyisovalerate	Delphinidin-3-rutinoside	Methylophiopogonanone a
2,4-di-tert-butylphenol	Dictamnine	Mulberrin
2,8-quinolinediol	Dihydropalmatine	N4-acetylsulfamethoxazole
2,8-quinolinediol sulfate	Dihydrosamidin	N-methylanthranilic acid
2-aminophenol	Quinaldic acid	N-methylpipecolate
2-hydroxyhippurate	Eleutheroside e	Pelargonin
2-methylpyrrolidine	Engeletin	Phenylacetylglutamine
2-oxindole-3-acetic acid	Enterolactone sulfate	Phenylalanine
2-oxo-1,2-dihydro-quinoline-3,4-dicarboxylic acid	Epigallocatechin	Pilocarpine
3,4-dihydrocoumarin	Equol sulfate	Pinoresinol 4-o-beta-d-glucopyranoside
3-acetylindole	Ergothioneine	Piperidine
3-phenylpropionate	Eriocitrin	Piperine
4-ethylcatechol sulfate	Ethyl gallate	Piperlotine a
4-ethylphenol	Fagomine	Pongamol
4-hydroxychalcone	Feruloyl putrescine	Propachlor
5,6,2'-trimethoxyflavone	Feruloyltyramine	Protocatechuic acid
5,7-dihydroxy-4'-methoxyflavanone 7-rutinoside	Gelomulide n	P-salicylic acid
5-butyl-3-methyl-7h-furo[3,2-g]chromen-7-one	Gerberinside	Pyrocatechol sulfate
5-hydroxyindole-3-acetic acid	Gingerol	

6,8-diprenylorobol	Glycitin	Quinate
6-ethyl-2,3,5-trimethyl-7h-furo[3,2-g]chromen-7-one	Gomisin h	Resveratrol 4'-methyl ether
6-methoxy-4-methyl-2h-chromen-2-one	Guaiacol sulfate	Rinderine
6-methoxybenzoxazolinone	Harman-3-carboxylic acid	Salidroside
6-o-acetyldaidzin	Harringtonine	Salsolinol
6-shogaol	Hesperidine	Schisandrin b
7,8-dihydrobiopterin	Histidine betaine	Schisandrin c
Aloin a	Homostachydrine	Schisanhenol
Aloperine	Hycanthone	Scoparone
Alpinetin	Indole-3-carboxyaldehyde	Stachydrine
Ancistrocladine	Indolelactic acid	Tephrosin
Apigenin 6,8-digalactoside	Isocoumarin	Tetrahydroepiberberine
Apigenin-7-o-glucuronide	Isoliquiritin apioside	Tetrahydropalmatine
Arctiin	Kaempferol-3-o-glucoside	Tetramethylscutellarein
Bavachin	Kaempferol-3-o-robinobioside	Thioproline
Bergenin	Kaempferol-7-o-beta-d-glucopyranoside	Thymol sulfate
Bracteatin	Ketoprofen	Trans-cinnamaldehyde
Brucine	Laudanosine	Vanillin acetate
Chrysophanol	Liriodenine	Venlafaxine
Cinnamaldehyde	Lobaric acid	Vincarine
Corydaline	Lumichrome	Viridiflorene
Corymboside	Luteolin 4'-o-glucoside	
**Cow milk**		
2-dehydro-o-desmethylangolensin	5-caffeoylquinic acid	Harmalol
2-hydroxyenterodiol	6”-o-malonyldaidzin	Harmane
3,3',4'5-tetrahydroxystilbene	6-o-malonylgenistin	Hesperetin 3,7-o-diglucuronide
3-aminosalicylic acid	7-hydroxysecoisolariciresinol	Homoveratric acid
3-caffeoylquinic acid	Aminohippuric acid	Hydroxyflavone

3-coumarin	Benzeneethanamine	Jasmonic acid
3-hydroxybenzoate	Biochanin a	Lariciresinol
3-hydroxyisovaleric acid	Cholic acid	Norathyriol
3-methoxy-4-hydroxyphenylglycol sulfate	Chrysin	O-desmethylangolensin
3-methylbenzamide oxime	Coumestrol	Phenol
3-phenylpropanoic acid	Cyanidin 3-o-xylosyl-rutinoside	Phenylacetamide
4,7-dihydroxy-3-methoxyisoflavan	Daidzin	Phenylacetic acid
4-hydroxy-3,4,5-trimethoxystilbene	Dihydroferulic acid 4-o-glucuronide	Pyrogallol
4-hydroxy-3-methylbenzoic acid	Enterodiol	Quercetin 3-o-rutinoside
4-hydroxybenzoate	Equol	Quercetin 3-o-xylosyl-glucuronide
4-hydroxyhippuric acid	Genkwanin	Salicyluric acid
4-methoxycinnamic acid	Gentisaldehyde	Urolithin b 3-o-glucuronide
5-(3'-methoxy-4'-hydroxyphenyl)-gamma-valerolactone	Gomisin m2	Vanylglycol
**Goat meat**		
(E)-1-cinnamoylpyrrolidine	Garcimangosone c	Nigakinone
8-hydroxypinoresinol 4-glucoside	Garcimangosone d	Pelargonidin 3-sophoroside
Aegle marmelos alkaloid c	Hexyl salicylic acid	Pyranodelphinin a
Amyl salicylate	Kaempferol 3-(2'''-sinapoylsophoroside) 7-cellobioside)	Squamosamide
Cis-caffeoyl tartaric acid	Licoagrone	Vignatic acid a
Deoxoisocucurbitacin d	Murrayanine	
**Goat milk**		
3,4-Dihydroxybenzoic acid	Coumaroyl quinic acid_a	Naringin
4-o-Caffeoylquinic acid	Coumaroyl quinic acid_b	Quercetin 3 glucoside
5-o-Feruloyl quinic acid	Fisetin	Quercetin 3-(6′′′′-ferulylsophorotrioside)
8,8′-Methylenebiscatechin	Gallocatechin	Salicin
Apigenin glucoside	Hydroquinone	Salicortin

Benzyl alcohol	Hyperin	Saligenin_b
Caffeoyl hexoside	Kaempferide	Saligenin_der_a
Catechol-hexoside (coumaric acid_hexoside)	Larycitrin-rhamnoside	Saligenin-der-b
Chrysoeriol rutinoside	Mandelonitrile	Syringic acid
Coumaric acid_hexoside	Methylbenzoate	
**Sheep milk**		
(-)-Epicatechin-(2a-7) (4a-8)-epicatechin 3-o-galactoside	Caffeic acid 4-o-glucoside	Malvidin 3-o-(6"-acetyl-glucoside)
1-Sinapoyl-2-feruloylgentiobiose	Caffeoyl glucose	Malvidin 3-o-(6"-acetyzl-galactoside)
24-Methylcholestanol ferulate	Caftaric acid	M-aminobenzoic acid
24-Methylcholesterol ferulate	Carnosol	M-coumaric acid
24-Methylenecholestanol ferulate	Coumarin	M-cresol
24-Methyllathosterol ferulate	Cyanidin 3,5-o-diglucoside	Methoxy phenylacetic acid
2-Hydroxy-2-phenylacetic acid	Cyanidin 3-o-sophoroside	Methyl jasmonate
2-Hydroxybenzoic acid	Cyanidin 3-o-xylosyl-rutinoside	Myricetin
2-Hydroxyphenylacetic acid	Dalbergin	Neohesperidin
2-Methoxy-6-(all-transoctaprenyl)phenol	Dihydrobiochanin a	o-Coumaric acid
2-Methylhippuric acid	Dihydrocaffeic acid	o-Cresol
3,4,5,4'-Tetramethoxystilbene	Dihydrocaffeic acid 3-o-glucuronide	Ortho-hydroxyphenylacetic acid
3,4-Dihydroxy-5-all-transdecaprenylbenzoate	Dihydroglycitein	p-Coumaroylquinic acid
3,4-Dihydroxyphenylacetic acid	Dihydro-p-coumaric acid	p-Cresol
3,4-Dihydroxyphenylglycol	Dimethoxy curcumin	Pelargonidin 3-o-(6"-p-coumaroyl-glucoside)
3,4-Hydroxybenzoic acid	Dimethyl matairesinol	Peonidin 3-o-rutinoside
3,7-o-Diglucoside3-cresotinic acid	Diosmetin	Phenylacetyl glycine
3-Hydroxybenzoic acid	D-viniferin	Phenylalanyl-alanine
3-Hydroxyphenylacetic acid	Elaidic acid	P-hydroxyphenylacetic acid
3-Methylfuran	Epicatechin	Protocatechuic aldehyde

3-p-Coumaroylquinic acid	Epigallocatechin	Protocatecuic acid
4-Aminobenzoic acid	Epigallocatechin gallate	Psoralen
4-Hydroxycinnamic acid	Eriodictyol	Pterostilbene
4-Hydroxyenterodiol	Estradiol	Quercetin 3-o-galactoside 7-o-rhamnoside
4-Hydroxyphenylacetic acid	Estragole	Quercetin 3-o-rhamnosyl-galactoside
4-p-Coumaroylquinic acid	E-viniferin	Quercetin 3-o-rutinoside
4-Vinylphenol	Galangin	Resacetophenone
5-Heneicosylresorcinol	Homovanillic acid	Resveratrol
5-Methoxyindoleacetate	Hydroxytyrosol	Sakuranetin
5-p-Coumaroylquinic acid	Hydroxytyrosol 4-o-glucoside	Sesamol
5-Tricosylresorcinol	Isopeonidin 3-o-rutinoside	Siringic acid
6-Hydroxyenterodiol	Isosakuranetin	Syringaldehyde
6-Methoxy-3-methyl-2-all-transdecaprenyl-1,4-benzoquinol	Isoxanthohumol	Thymol
7,3',4'-trihydroxyflavone	Juglone	Tyrosol
Anethole	Kaempferol	Vanillin
Arctigenin	Kaempferol 3-o-sophoroside	Xanthohumol
Benzyl methyl sulfide	Kampferol	
**Beef, bison**		
2,6-dihydroxybenzoic acid	Benzoate	Phenol sulfate
3-Hydroxyhippurate	Benzoylcarnitine	Salicylate
4-Ethylphenyl sulfate	Catechol sulfate	
4-Methylcatechol sulfate	p-Cresol sulfate	
**Beef, buffalo milk, cow milk, goat milk, bison**		
N-Cinnamylglycine		
**Beef, buffalo milk, cow milk, goat milk, sheep meat, sheep milk**		
Daidzein		
**Beef, buffalo milk, cow milk, goat milk, sheep milk**		

4-Hydroxybenzoic acid	p-Coumaric acid	
**Beef, buffalo milk, cow milk, goat milk, sheep milk, bison**		
Hippuric acid		
**Beef, sheep milk**		
Rutin		
**Beef, goat milk**		
Methyl syringate		
**Beef, cow milk**		
Dimethyl sulfone	Gardenin b	Hordenine
Phenylacetylglycine		
**Beef, cow milk, goat milk, sheep meat**		
Enterolactone	Matairesinol	Theobromine
**Beef, cow milk, goat milk, sheep meat, sheep milk**		
Genistein	Glycitein	
**Beef, cow milk, sheep milk**		
Gallic acid	Vanillic acid	Formononetin
**Buffalo milk, cow milk, goat milk, sheep milk**		
1-Hydroxylamino-2-phenylethane	3-Hydroxycinnamic acid	4-Methylcatechol
1-o-Vanillyl-beta-d-glucose)	3-Hydroxyphenylurea	4-Tert-butylbenzoic acid
2-(4-Hydroxyphenyl) ethanol	3-Methoxytyramine	Catechol
2,6-di-tert-butyl-4-(hydroxymethyl) phenol	4-Acetylaminobenzoic acid	Hydroumbellic acid
2-Amino-5-nitrobenzoic acid	4-Hydroxybenzaldehyde	Hydroxyphenyllactic acid
2-Butyl-3-(4-hydroxybenzoyl) benzofuran	4-Hydroxyhippurate	Shikimic acid
2-Furoylglycine	4-Hydroxyquinoline	Synephrine

3-Aminobenzamide	4-Methoxyphenol	
**Cow milk, goat milk**		
Benzaldehyde	Salicylic acid	
**Cow milk, goat milk, sheep milk**		
Apigenin	Naringenin	Theophylline
Kaempferol	Quercetin	
**Cow milk, sheep milk**		
4-Methoxybenzaldehyde	Ferulic acid	Catechin
5-(3',5'-dihydroxyphenyl)-gamma-valerolactone 3-o-glucuronide	Luteolin	Chlorogenic acid
Benzoic acid	Piceatannol	Esculetin
Delphinidin 3-o-rutinoside	Rosmarinic acid	Hesperidin
Ellagic acid	Schisandrin	

Supplemental Table S2. Study-level data extraction for 39 studies, including sample type; country of origin; experimental group (e.g., breed, fresh vs. conserved forage, short- vs. long-term pasture, month of year, commercial samples, milk-fat content); measured phenolics (relevant compounds extracted from metabolomics datasets); concentrations (where reported, with n per group); analytical platform; and references.

Sample Type	Origin (Country/Breed/Feed)	Phenolics			Reference
		Total Concentration	Measured Compounds	Numbers via equipment	
Cow milk	Finland, Breed: NA, Feed: NA	ng/mL 414.5 (Organic); 61.2 (Conventional)	Equol; formononetin	2 via HPLC-DAD	Hoikkala et al. [74]
Goat Milk; Cow Milk; Cattle Meat; Sheep Meat	UK, Breed: NA, Feed: NA	ng/mL 90 (cow milk); 10 (goat milk); 150 (beef); 60 (sheep meat)	Daidzein; genistein; enterolactone; matairesinol; glycitein	5 via HPLC-QQQ-MS (QTRAP-4000)	Kuhnle et al. [108]
Cow Milk	Norway, Breed: Norwegian Red Breed, Feed:White and red clover, ryegrass swards, ± concentrate feed (oats+hay)	ng/mL 85 (white clover); 125 (white clover with concentrate); 104 (red clover); 118 (red clover with concentrate)	Formononetin; daidzein; equol; biochanin a; genistein; prunetin; secoisolariciresinol; matairesinol; enterodiol; enterolactone	10 via HPLC-QQQ-MS (Micromass)	Steinshamn et al. [109]
Cow Milk	Denmark, Breed: Holstein, Feed: lucerne, white clover, red clover, chicory, perennial ryegrass, Calibre, Sameba, Tivoli	ng/mL 655 (May); 460 (June); 63 (White clover); 325 (Red clover); 91 (Lucerne); 78 (Chicory)	Chrysin; biochanin a; equol; glycitein; daidzein; enterodiol; enterolactone; naringenin; formononetin	9 via HPLC-DAD	Andersen et al. [73]
Cow Milk	Finland, Breed: Finnish Ayrshire Breed, Feed: Grass silage, red clover silage, barley–oats mix, rapeseed expellers	ng/mL 171–287 (Grass silage); 458–643 (Red clover silage)	Equol	1 via HPLC-DAD	Mustonen et al. [75]

Cow Milk	Czech Republic, Breed: Holstein, Feed: Extruded rapeseed cake/full-fat soybeans, maize silage, barley, oat, meadow hay	ng/mL<br>50 (Rapseed); 108 (Soybeans)	Daidzein; equol; genistein	3 via HPLC-QIT-MS (Thermo Finnigan)	Třináctý et al.[76]
Cow Milk	France,<br>Breed: Montbéliarde and Tarentaise<br>Feed: Concentrate rich+cocksfoot hay; Maize silage; Rryegrass silage; Ryegrass hay; Grassland hay, Grassland pasture	ng/mL<br>813 (Concentrate rich+cocksfoot hay);<br>1759 (Maize silage);<br>1196 (Rryegrass silage);<br>1841 (Ryegrass hay);<br>1094 (Grassland hay);<br>6877 (Grassland pasture)	4-Hydroxybenzoic acid; phenylacetic acid; catechol; hippuric acid; benzoic acid; ferulic acid; benzaldehyde; quercetin; luteolin; apigenin	10 via HPLC-DAD	Besle et al.[113]

Cow Milk	Sweden, Breed: Swedish Red, Feed: Short-term Pasture: Timothy+red clover hay, Long-term Pasture: Timothy + meadow fescue + perennial ryegrass + white clover hay, 2-cut Red Clover-Grass: red clover (spring growth + 1 summer regrowth)+timothy + meadow fescue silage, 3-cut Red Clover-Grass: red clover (spring growth + 2 summer regrowth)+timothy + meadow fescue silage, 2-cut Birdsfoot Trefoil-Grass: birdsfoot trefoil (spring growth and 1 summer regrowth cut)+timothy	µg/kg<br>801 (Short-term pasture); 237 (Long-term pasture); 1659 (Silage of 2-cut red clover-grass); 433 (3-cut red clover-grass); 403 (2-cut birdsfoot trefoil-grass)	Equol; genistein; daidzein; biochanin a; formononetin; secoisolariciresinol; enterolactone; prunetin; matairesinol; coumestrol; enterodiol	11 via HPLC-QQQ-MS (Quattro)	Höjer et al. [110]

Cow Milk	Norway, Breed: Norwegian Red, Feed: Organic Systems (Grasses moderate to lower & legumes: higher & others: higher) Conventional Systems (Grasses: higher to moderate & legumes: lower & others: lower, Short-Term) Grassland (Grasses: moderate & legumes: higher & other: higher, Long-Term Grassland): Grasses: lower & legumes: higher & other: higher, Where Grasses (Poaceae), such as timothy, meadow fescue, perennial ryegrass, smooth meadowgrass, rough meadowgrass, and common couch; legumes (Fabaceae), including red clover and white clover; whereas, other botanical families, such as northern dock, dandelion, common sorrel, creeping buttercup	μg/kg<br><br>203 (Indoor); 197 (Outdoor); 256 (Short-term grassland); 146 (Long-term grassland); 542 (Organic); 261 (Conventional)	Biochanin A; daidzein; equol; genistein; matairesinol; secoisolariciresinol; enterodiol; enterolactone; formononetin; prunetin	10, via HPLC-QQQ-MS (Micromass)	Adler et al. [114]

Goat Milk	Italy, Breed: Girgentana, Feed: Sulla fresh forage: Sulla fresh forage+barley meal, barley meal+mixed hay (berseem clover, Lolium, Reed canary grass), Farming practices: conventional	µg GAE/mL<br>57 (Fresh Sulla); 56 (Sulla+Barley meal); 49 (Hay+Barley meal)	-		Di-Trana et al. [62]
Cow Milk	Canada, Breed: N/A, Feed: N/A	µM<br>67 (Skim milk), 65 (1% fat milk); 69 (2% fat milk); 72 (3.25% fat milk)	Hippuric acid	1 via NMR	Forouta n et al. [86]
Goat Milk	Mexico, Breed: Alpine, Feed: Control diet: Alfalfa hay and corn silageFree-Range diet: Prosopis laevigata, wattles, thorntrees, whistling thorns, hackberries, prickly pear cactus, and grasses	µg GAE/mL<br>Alfalfa Hay and Corn Silage=65<br>Free-Rangeland Grazing=88<br>Unpasteurized=89<br>Pasteurized=64.15<br>Dry=87<br>Rainy=66	-	-	Chávez-Servín et al. [61]

Sheep Meat	Italy, Breed: Valle del Belice, Feed: Control Diet: No durum wheat bran, Wheat bran Diet: 20% of durum wheat bran. With alfalfa pelleted hay, faba bean and barley grains coarsely ground meal	µg GAE/g<br>ad libitum, Control (n=14) 0.190; ad libitum, 20% Wheat Bran (n=14) 0.167; Restricted, Control (n=10) 0.287; Restricted, 20% Wheat Bran (n=9) 0.416	-	-	Di-Grigoli et al., [59]
Sheep Milk; Goat Milk	Switzerland, Breed: Saanen goats and Friesian sheep, Feed: Control diet: Sugar beet pulp, Grapeseed extract-based diet: Grapeseed extract, With Maize, barley, wheat, wheat bran, mill by-products, molasses, wheat starch and Hay from mixed swards (grass, legumes, and herbs)	µg GAE/mL<br>Sheep<br>33 (CON); 38 (Grape seed extract supplemented)<br>Goat<br>30 (CON); 38 (Grape seed extract supplemented)	-	-	Leparmarai et al. [64]
Cow Milk	UK, Breed: NA, Feed: NA	ng/mL<br>131 (CON); 484 (ORG); 131 (FRG)	Equol; secoisolariciresinol; matairesinol; lariciresinol; enterolactone; enterodiol; daidzein; genistein; glycitein; formononetin; naringenin; coumestrol	12 via HPLC QQQ-MS (AB QTRAP-5500)	Nørskov et al. [123]

Goat Meat	China, Breed: Lubei white, Boer, and Jining grey, Feed: Peanut hay, Corn, Soybean meal, and Cottonseed meal	-	Kaempferol 3-(2'''-sinapoylsophoroside) 7-cellobioside); 8-hydroxypinoresinol 4-glucoside; Pelargonidin 3-sophoroside; Cis-Caffeoyl tartaric acid; garcimangosone C & D; licoagrone; pyranodelphinin A; amyl salicylate; hexyl salicylic acid; deoxoisocucurbitacin D; vignatic acid A; Squamosamide; murrayanine; nigakinone; (E)-1-cinnamoylpyrrolidine; ceanothine E; solanocardinol: aegle marmelos alkaloid C	19 via UPLC-QToF-MS (Agilent Xevo G2-XS)	Wang et al. [80]
Sheep Milk	Italy, Breed: Sarda, Feed: Natural pasture control, Natural pasture with Selected pasture: Oat, barley and crimson clover, With Giant Fennel, Deadly Carrot, Branched Asphodel, Common Bracken, Sea Squill, and White Clover	-	Hippuric acid	1 via GC-MS (HP- 5973)	Scano et al. [84]

Cow Milk	Italy, Breed: NA, Commercial samples	-	2-Dehydro-O-desmethylangolensin; Delphinidin 3-O-rutinoside; Quercetin 3-O-rutinoside; Cyanidin 3-O-xylosyl-rutinoside; 2-Hydroxyenterodiol; Gomisin M2; Schisandrin; Pyrogallol; Norathyriol; Urolithin B 3-O-glucuronide; 5-(3'-Methoxy-4'-hydroxyphenyl)-gamma-valerolactone; Dihydroferulic acid 4-O-glucuronide; 5-Caffeoylquinic acid; Rosmarinic acid; 5-(3',5'-dihydroxyphenyl)-gamma-valerolactone 3-O-glucuronide; 3,3',4'5-Tetrahydroxystilbene; Cholic acid	18 via UPLC-quadrupole-Orbitrap-MS (Q Exactive™ Focus)	Rocchetti et al. [124]

Goat Milk	Italy, Breed: Saanen, Feed: Control diet: N/A, Olive leaves-based diet: Olive leaves, With Corn meal, soya flour, barley meal, and beet pulp	µg GAE/mL<br>29.75 (CON); 39.53 (OL)<br>µg/mL<br>0.451 (OL)	3,4-Hydroxybenzoic acid; caffeic acid; catechin; chlorogenic acid; cinnamic acid; diosmetin; ferulic acid; gallic acid; hydroxytyrosol; kampferol; luteolin; myricetin; o-coumaric acid; p-coumaric acid; quercetin; rosmarinate; rutin; tyrosol; vanillic acid	19 via UPLC QQQ-MS (AB QTRAP-4500)	Ianni et al. [63]

Cow Milk	Kenya Breed: Somali (camel), East African Boran and Guernsey (cross bread cattle), small east African shorthorn zebu (local cattle), Feed: RS Site: Woody Vegetation: Whistling Thorn, Acacia nilotica, and Acacia seyal, Woody Vegetation: Whistling Thorn, Acacia nilotica, and Acacia seyal, Herbaceous Layer: Hyparrhenia papillipes, Kangaroo Grass, TP Site: Woody Vegetation: Shepherd's Tree and Common Spike-thorn, Herbaceous Layer: Pennisetum and Hyparrhenia papillipes, Herbs: Barleria delamerei (dominant in both sites and seasons)	µg GAE/mL<br>All treatment results RS+Crossbred+Control 44.6; RS+Crossbred+Urea 46.7; RS+Pokot+Control (n=6) 58.3; RS+Pokot+Urea (n=5) 42.9; TP+Crossbred+Control (n=6) 28.2; TP+Crossbred+Urea (n=5) 25.3; TP+Pokot+Control (n=6) 28.1; TP+Pokot+Urea (n=5) 25.0	,	-	Leparmarai et al. [65]

Sheep Milk	Italy Breed: Sarda ewes, Feed: Control diet: total mixed ration, soybean hulls, Soybean hulls diet: total mixed ration, soybean hulls, cocoa husks, Cocoa husks diet: total mixed ration, cocoa husks	-	Theobromine; 3,4-dihydroxy-5-all-transdecaprenylbenzoate; 6-methoxy-3-methyl-2-all-transdecaprenyl-1,4-benzoquinol; 2-methoxy-6-(all-transoctaprenyl)phenol	4 via UHPLC-IMS-TOF-MS (Agilent 6560)	Manis et al. [121]
Cow Milk	China, Breed: Holstein, Feed: CON group: total mixed ration<br>Treatment group: total mixed ration and Perilla frutescens leaf	-	Daidzin; ononin; 6”-O-malonyldaidzin; rosmarinate; phenylacetic acid; genkwanin; theophylline; 3-coumarin; phenylacetamide; vanylglycol; 3-hydroxycinnamic acid; hydroxyflavone; 3-hydroxybenzoate; 3-hydroxyisovaleric acid	14, UHPLC-IMS-TOF-MS (Agilent 6560)	Wang et al. [19]
Sheep Milk	Italy, Breed: crossbreed, Feed: Control: Beet pulps, Treatment: Grape Pomace, With triticum, wheat, corn, barley, sunflower, soya and white sorghum	µg GAE/mL<br>26 (CON); 30 (10% GPS)	-	-	Bennato et al. [60]

Cow Milk	China, Breed: Holstein, Feed: Control: low calcium propionate, Treatment: low to high calcium propionate, With sprouting corn bran, stem-flaked corn, cottonseed, pelleted beet pulp, wet brewer grains, alfalfa, oat hay and corn silage	-	3-Methylbenzamide oxime; 3-phenylpropanoic acid; 4-hydroxybenzoate; equol; quercetin; daidzein; harmane; phenol; enterodiol; gentisaldehyde; benzeneethanamine; salicylic acid; genistein; salicyluric acid; 3-methoxy-4-hydroxyphenylglycol sulfate; 4-hydroxy-3-methylbenzoic acid; benzoic acid; dimethyl sulfone; harmalol; kaempferol; vanillic acid; aminohippuric acid; gallic acid; hippuric acid; hordenine; jasmonic acid; trans-cinnamate, phenylacetylglycine; 2-furoylglycine; 3-aminosalicylic acid; 4-methoxycinnamic acid	31 via UPLC- Q-TOF/MS (AB-5600+)	Zhang et al. [125]

Sheep Milk	Italy, Breed: crossbreed, Feed: Control: Beet pulps, Treatment: Grape Pomace, With triticum, wheat, corn, barley, sunflower, soya and white sorghum	ng/ml 1280 (CON); 1800 (GPS)	Caftaric acid; caffeic acid; chlorogenic acid; ellagic acid; ferulic acid; gallic acid; p-coumaric acid; protocatecuic acid; rosmarinic acid; siringic acid; trans-cinnamic acid; vanillic acid; 4-hydroxybenzoic acid; naringenin; epigallocatechin; catechin; epicatechin; epigallocatechin gallate; kaempferol; luteolin; quercetin	21 via HPLC QQQ-MS (AB QTRAP-4500)	Bennato et al. [54]
Goat Milk	Italy, Breed: Saanen, Feed, Control: concentrate, Treatment: low to high dose of spent coffee grounds, With haylage, soybean meal, beet pulp	-	Theobromine, theophylline; 8,8′-methylenebiscatechin; quercetin 3-(6-ferulylsophorotrioside)	4 via UHPLC-IMS-TOF-MS (Agilent 6560)	Casula et al. [127]

Goat Milk	Greece, Breed: Damascus, Indigenous Greek, Alpine, Damascus, Cross breeds (Indigenous Greek, Alpine, Damascus), Feed: Crops: Corn, straw, corn stalks, oats, beans, alfalfa, Grasses: Grassland grasses, Trees/Shrubs: Oak, rowan, low bushes (kermes oak), tree spurge, Herbs/Legumes: Wild vetch	µg GAE/mL 1100 (March); 1200 (April); 1150 (May); 1100 (June); 1180 (July); 1240 (August); 1390 (September)	-	-	Kasapidou et al. [43]
Cattle Meat	USA, Breed: Red Angus steer, Feed: Control: orchard grass hay, dry corn, high-moisture corn, Grass diet: meadow fescue, red clover, timothy grass, alfalfa, white clover, birdsfoot trefoil, chicory, orchard grass, and dandelion, Grape pomace diet: orchard grass hay, dry corn, high-moisture corn with grapeseed extract	µg/g 155.6 (CON); 159.0 (GPS); 217.1 (Grass)	4-Ethylphenol; hippuric acid; p-coumaric acid; caffeic acid; coixol; pyrocatechol sulfate; 4-hydroxybenzoic acid; vanillic acid; gallic acid; ethyl gallate	10 via UPLC-QQQ (AB QTRAP-7500)	Krusinski et al. [5]

Goat Milk	Israel, Breed: Damascus crossbred, Feed: Oats hay, Vetch hay, and willow trees silage	-	Methylbenzoate; salicin; 3,4-dihydroxybenzoic acid; caffeic acid; catechin; chlorogenic acid; esculetin; fisetin; genistein; apigenin; hesperidin; kaempferol; naringenin; naringin; 4-hydroxybenzoic acid_der; 4-o-caffeoylquinic acid; 5-o-feruloyl quinic acid; apigenin glucoside; caffeoyl hexoside; chaenomeloidin; chrysoeriol rutinoside; coumaric acid_hexoside; coumaroyl quinic acid_a; coumaroyl quinic acid_b; gallocatechin; hyperin; kaempferide; kaempferol hexoside; larycitrin-rhamnoside; salicortin; saligenin_b; syringic acid; quercetin; quercetin 3 glucoside; mandelonitrile; hydroquinone; hippuric acid; saligenin-der-b;	44 via LC-TOF-MS	Landau et al. [128]

			salicylic acid; 4-hydroxybenzoic acid; methyl syringate; benzaldehyde; benzyl alcohol; saligenin_der_a; benzaldehyde		

Sheep Milk	Italy, Breed: Sarda, Feed: Natural pasture, hay, concentrate, and standing hay Natural pasture: Subterranean Clover, Black Bog-rush, Small Hop Clover, Soft Brome, Little Bur Clover, Compact Brome, Sea Barley, Annual Bluegrass, Calepina, Gaudinia, Perennial Ryegrass, Wild Oat, Annual Vernal Grass, Barley Grass, Bearded Barley, Crested Dog's-tail, Ribwort Plantain, Bearded Fescue, Field Madder, Yanninicum Clover, Hairy Buttercup, Black-grass, Sticky Mouse-ear Chickweed, Blue Canary Grass, Italian Fescue, Tuberous Hawk's-beard, Rat's-tail Fescue, Burr Medic, Couch Grass, Spanish Salsify, Rough Dog's-tail, Dove's-foot Crane's-bill, Corn Chamomile, Bird's-foot	-	Carnosol; 3,4,5,4'-tetramethoxystilbene; caffeic acid 4-o-glucoside; caffeoyl glucose; dimethyl matairesinol; dihydrocaffeic acid 3-o-glucuronide; rosmarinic acid; elaidic acid; resveratrol; vanillic acid; juglone; 4-hydroxybenzaldehyde; hippuric acid; m-aminobenzoic acid; p-aminobenzoic acid; 3-methylfuran; sakuranetin; 3-p-coumaroylquinic acid; 4-p-coumaroylquinic acid; 5-p-coumaroylquinic acid; dimethoxy curcumin; p-coumaroylquinic acid; p-anisaldehyde; 7,3',4'-trihydroxyflavone; apigenin; eriodictyol; galangin; genistein; dihydro-p-coumaric acid; methoxy phenylacetic acid; cinnamic acid;	117 via UHPLC-Q-TOF-MS (Agilent 6550)	Rocchetti et al. [56]

	Trefoil, Harding Grass, Bluegrass, Medusahead, Shepherd's Purse, Skeletonweed, Brighteyes, Weak Bluegrass, Toad Rush, and Compact Brome.		pelargonidin; phenylalanyl-alanine; 2-methylhippuric acid; phenylacetyl glycine; benzyl methyl sulfide; m-cresol; o-cresol; p-cresol; estradiol; 4-hydroxycinnamic acid; m-coumaric acid; p-coumaric acid; psoralen; cyanidin 3-o-xylosyl-rutinoside; coumarin; 2-hydroxybenzoic acid; 3-hydroxybenzoic acid; 4-hydroxybenzoic acid; 5-(3',5'-dihydroxyphenyl)-gamma-valerolactone 3-o-glucuronide; protocatechuic aldehyde; catechol; sesamol; thymol; 4-vinylphenol; pterostilbene; 5-methoxyindoleacetate; methyl jasmonate; (-)-epicatechin-(2a-7) (4a-8)-epicatechin 3-o-galactoside; 1-sinapoyl-2-feruloylgentiobiose; 3,4-dihydroxyphenylglycol		

			; esculetin; hydroxytyrosol; arctigenin; isoxanthohumol; xanthohumol; hydroxytyrosol 4-o-glucoside; piceatannol; dihydrocaffeic acid; homovanillic acid; syringaldehyde; 24-methylcholesterol ferulate; 24-methylenecholestanol ferulate; 24-methyllathosterol ferulate; 4-hydroxyenterodiol; 6-hydroxyenterodiol; 3,4-dihydroxyphenylacetic acid; anethole; estragole; schisandrin; peonidin 3-o-(6"-p-coumaroyl-glucoside); malvidin 3-o-(6"-acetyzl-galactoside); malvidin 3-o-(6"-acetyl-glucoside); isopeonidin 3-o-rutinoside; peonidin 3-o-rutinoside; hesperidin; neohesperidin; 24-methylcholestanol		

			ferulate; benzoic acid; kaempferol 3-o-sophoroside; kaempferol 3,7-o-diglucoside; quercetin 3-o-galactoside 7-o-rhamnoside; quercetin 3-o-rhamnosyl-galactoside; 5-tricosylresorcinol; quercetin 3-o-rutinoside; 5-heneicosylresorcinol; cyanidin 3-o-sophoroside; cyanidin 3,5-o-diglucoside; delphinidin 3-o-rutinoside; d-viniferin; e-viniferin; dalbergin; dihydrobiochanin a; dihydroglycitein; formononetin; isosakuranetin; 2-hydroxy-2-phenylacetic acid; 2-hydroxyphenylacetic acid; 3-cresotinic acid; 3-hydroxyphenylacetic acid; 4-hydroxyphenylacetic acid; ortho-hydroxyphenylacetic acid; p-		

			hydroxyphenylacetic acid; resacetophenone; vanillin		
Cow Milk	Itlay, Breed: NA, Commercial samples, high-moisture corn with legume silage vs. compound feed	-	3-Caffeoylquinic acid; 4,7-Dihydroxy-3-methoxyisoflavan; 4-Hydroxy-3,4,5-trimethoxystilbene; 4-Hydroxyhippuric acid; 6-O-Malonylgenistin; 7-Hydroxysecoisolariciresinol; trans-Cinnamic acid; Ellagic acid; Enterolactone; Equol; Formononetin; Gardenin B; Hesperetin 3,7-O-diglucuronide; Hippuric acid; Homoveratric acid; O-Desmethylangolensin; 4-Methoxybenzaldehyde; Piceatannol; Quercetin 3-O-xylosyl-glucuronide; Vanillic acid.	20 via UPLC-quadrupole-Orbitrap-MS (Q Exactive™ Focus)	Rocchetti et al. [126]

Cow milk	Denmark, Breed: Danish Holstein, Danish Jersey, cross-breed or mixes of these, Feed: NA	ng/mL<br>108 (CON); 305 (ORG); 493 (BD)	Equol; formononetin; naringenin; matairesinol; lariciresinol; secoisolariciresinol; enterodiol; daidzein; enterolactone; genistein	10 via LC-Qtrap (Ab QTRAP-550)	Róin et al. [119]
Cow Milk; Goat Milk	Hungary, Breed: Saanen goat and Jersey cattle, Feed: NA	µg GAE/mL<br>49.0 (Saanen goat); 48.3 (Jersey cow); 47.9 (Holstein cow); 42.0 (Simmental cow)	-	-	Sik et al. [66]

Bison Meat	USA, Breed: NA, Feed: Pen finished diet: Pasture finished diet: Sand bluestem, prairie sandreed, switchgrass, prairie cordgrass, little bluestem, yellow Indian grass, blue grama, buffalo grass, sand muhly, red threeawn, sand dropseed, sand love grass, tumblegrass, lowoutgrass, needle and thread, western porcupine grass, green needle grass, june grass, western wheatgrass, threadleaf sedge, sun sedge, cheatgrass, annual sunflower, stiff sunflower, prickly poppy, bush morning glory, sweet clover, sand milkweed, American vetch, milkvetches, locoweeds, silver scurfpea, beardtongue, purple prairie clover, dotted blazing star, wavyleaf thistle, western ragweed, spiderwort, prairie coneflower, rocky mountain bee	-	4-Ethylphenylsulfate; catechol sulfate; 3-hydroxyhippurate; cinnamoylglycine; 2,6-dihydroxybenzoic acid; hippurate; phenol sulfate; benzoylcarnitine; p-cresol sulfate; 4-methylcatechol sulfate; n-methylpipecolate; benzoate; salicylate	13 via via UPLC-quadrupole-Orbitrap-MS (Q Exactive™ Focus)	Van Vliet et al. [6]

	plant, sand verbena, blue vervain, yucca, sand cherry, chokecherry, and snowberry.				
Cattle Meat	USA, Breed: Angus, Feed: Grass finished: rangeland grasses, forbs, and shrubs, Grain-finished: corn-based total mixed ration	-	Hippurate; 3-Hydroxyhippurate; Benzoylcarnitine; 4-Ethylphenylsulfate; Phenol sulfate; n-Methylpipecolate; Catechol sulfate; p-Cresol sulfate; 2,3-Dihydroxyisovalerate; 2,6-Dihydroxybenzoic acid; Guaiacol sulfate; Cinnamoyl glycine; Dimethyl sulfone; Salicylate; Benzoate; piperidine and 4-methylcatechol sulfate	17 via UPLC-quadrupole-Orbitrap-MS (Q Exactive™ Focus)	Evans et al.[4]

Sheep Milk; Goat Milk; Buffalo Milk; Cow Milk	China, Breed: Xinong Saanen dairy goats, Holstein cows, East Friesian dairy sheep, commercial farm buffaloes, Feed: Corn, soybean meal, bran, rapeseed meal	-	4-Tert-butylbenzoic acid; n-cinnamylglycine; 4-hydroxyhippurate; 4-methoxyphenol; 4-methylcatechol; 2-amino-5-nitrobenzoic acid; daidzein; 4-hydroxyquinoline; 3-aminobenzamide; p-coumaric acid; 4-hydroxybenzaldehyde; caffeic acid; 4-acetylaminobenzoic acid; hippuric acid; 2-(4-hydroxyphenyl) ethanol; 2,6-di-tert-butyl-4-(hydroxymethyl) phenol; 1-o-vanillyl-beta-d-glucose); 2-furoylglycine; shikimic acid; hydroxyphenyllactic acid; 4-hydroxybenzoic acid; hydroumbellic acid; 3-hydroxycinnamic acid; catechol; 3-hydroxyphenylurea; 1-hydroxylamino-2-phenylethane; 2-butyl-3-(4-hydroxybenzoyl)	30 via UPLC-QQQ-QTrap (AB)	Zhang et al. [122]

			benzofuran; synephrine; 3-methoxytyramine; zereno		

Cow Milk and Goat Milk	Italy, Breed: NA, Commercial	ng mL⁻¹<br>Cow milk<br>Microfiltered semi-skimmed (n=2) 13230 Semi-skimmed pasteurized (n=2) 12462 Semi-skimmed UHT (n=2) 13058 Whole lactose-free UHT (n=2) 14024 Whole Pasteurized (n=2) 16332 Whole UHT (n=2) 69676 Goat Milk<br>Semi-skimmed UHT (n=2) 26755 Whole UHT (n=2) 15098	Dihydrogenistein-7-glucuronide; Daidzein-7-glucuronide; O-Desmethylangolensin-sulfate; Hydroxyequol-glucuronide; Hydroxyequol-sulfate; Equol-7-glucuronide; Equol-7-sulfate; Equol-4′-sulfate; 5-(3′,4′,5′-Trihydroxyphenyl)-γ-valerolactone; 5-(3′/4′-Hydroxyphenyl)-γ-valerolactone-sulfate; 5-Phenyl-γ-valerolactone-4′-sulfate; 5-Phenyl-γ-valerolactone-3′-sulfate; Enterolactone-glucuronide; Enterolactone-sulfate; Enterolactone; Enterodiol-sulfate; Dihydroxy-urolithin-glucuronide (urolithin C-glucuronide); 3-Hydroxy-urolithin-8-glucuronide (urolithin A-glucuronide); 3-Hydroxy-urolithin-8-sulfate (urolithin A-sulfate); Urolithin-3-glucuronide (urolithin	26 via UPLC-QQQ-MS (TSQ Vantage)	Agulló et al.[49]

			B-glucuronide); Urolithin-3-sulfate (urolithin B-sulfate); 3′-Hydroxyhippuric acid; Hippuric acid; Benzene-1/2-ol-sulfate (isomer 1, catechol-sulfate 1); Benzene-1/2-ol-sulfate (isomer 2, catechol-sulfate 2).		

Cattle Meat	USA, Breed: Angus, Feed: Grass finished: tall fescue, red clover, orchard grass, crabgrass, tall fescue, orchard grass, plantain, white cloverat, ryegrass, plantain, and chicory, Grain-finished: corn-based total mixed ration	-	Hippurate; 2-hydroxyhippurate; 3-hydroxyhippurate; Catechol sulfate 4-ethylcatechol sulfate; 4-methylcatechol sulfate; 4-ethylphenyl sulfate; P-cresol sulfate 3-phenylpropionate; Piperidine; Harman-3-carboxylic acid; Gluconate Cinnamoylglycine; Enterolactone; Enterolactone sulfate; Equol sulfate; Ergothioneine; Fagomine; Quinate; Histidine betaine; Homostachydrine; Piperine; Stachydrine; Thymol sulfate; N-methylpipecolate; 2,6-dihydroxybenzoic acid; 1,3-propanediol; O-sulfo-tyrosine; Dimethyl sulfone; Glycerol 2-phosphate; Thioproline; 2,8-quinolinediol sulfate; 2,4-di-tert-butylphenol	33 via UPLC-quadrupole-Orbitrap-MS (Q Exactive™ Focus)	Ahsin et al. [12]

Cattle Meat	USA, Breed: NA, Commercial samples Ground Beef and Plant based meat	-	Pilocarpine; Rinderine; Liriodenine; Magnoflorine; Harringtonine; Vincarine; Aloperine; Tetrahydroepiberberine; Dihydropalmatine; Tetrahydropalmatine; Corydaline; Brucine; Chrysophanol; Aloin A; Propachlor; 2-Aminophenol; N4-Acetylsulfamethoxazole; N-Methylanthranilic Acid; Methyl syringate; Bergenin; p-Salicylic acid; Protocatechuic acid; Ketoprofen; Hordenine; Cyproconazole; Vanillin acetate; 1,2,4-Trimethoxy-5-propenylbenzene; Venlafaxine; (R)-(-)-Phenylephrine; Dopamine; Gingerol; Feruloyltyramine; 6-Shogaol; Magnolin; Pinoresinol 4-O-beta-D-glucopyranoside; Arctiin; Eleutheroside E; Phenylacetylglycine; Cinnamoylglycine;	117 via UPLC-Tof-MS (Agilent-6545)	Hernandez et al. [57]

			Phenylacetylglutamine; Phenylalanine; Ergothioneine; Salidroside; Coumaric acid 4-O-glucoside; Theobromine; 3-Acetylindole; Indole-3-carboxyaldehyde; 5-Hydroxyindole-3-acetic acid; 2-Oxindole-3-acetic acid; Indolelactic acid; Laudanosine; Ancistrocladine; Gelomulide N; Lumichrome; 7,8-Dihydrobiopterin; Methyl 3-(4-hydroxy-6-methyl-2-oxo-2H-pyran-3-yl)-3-phenylpropanoate; 2-Methylpyrrolidine; Viridiflorene; Dictamnine; Quinaldic acid; 2-oxo-1,2-dihydro-quinoline-3,4-dicarboxylic acid; 1-methyl-2-undecylquinolin-4-one; 2,8-Quinolinediol; Salsolinol; 1-(2-hydroxy-3-methyl)-butyl-hydrocotarnine; Hycanthone; trans-		

			Cinnamaldehyde; Scoparone; 6-o-Acetyldaidzin; 3,4-Dihydrocoumarin; Bracteatin; Cinnamaldehyde; Piperlotine A; Feruloyl putrescine; 6-methoxy-4-methyl-2H-chromen-2-one; Gerberinside; 6-ethyl-2,3,5-trimethyl-7H-furo[3,2-g]chromen-7-one; 5-butyl-3-methyl-7H-furo[3,2-g]chromen-7-one; Dihydrosamidin; Lobaric Acid; Bavachin; Mulberrin; Corymboside; Apigenin 6,8-digalactoside; Apigenin-7-O-glucuronide; Luteolin 4'-O-glucoside; Kaempferol-7-O-beta-D-glucopyranoside; Kaempferol-3-O-glucoside; Engeletin; Isoliquiritin apioside; Kaempferol-3-O-robinobioside; Rutin; Pelargonin; Delphinidin-3-		

			rutinoside; Eriocitrin; 5,7-Dihydroxy-4'-methoxyflavanone 7-rutinoside; Hesperidine; Delphinidin; Alpinetin; 5,6,2'-Trimethoxyflavone; Tetramethylscutellarein; Gardenin B; Methylophiopogonanone A; Isocoumarin; 6,8-Diprenylorobol; Glycitin; Daidzin 6'-O-malonate; Formononetin; Tephrosin; Pongamol; 4-Hydroxychalcone; Resveratrol 4'-Methyl Ether; Schisandrin C; Schisandrin B; Schisanhenol; Gomisin H; Epigallocatechin		

Where NA: Information not available or not reported, GAE: Gallic Acid Equivalents, CON: Control diet, ORG: Organic, FRG: Free-range, GPS: Grape Pomace supplementation, OL: Olive leaves and BD: Biodynamic
